# A conserved uORF in the *ilvBNC* mRNA of *Corynebacterium* species regulates *ilv* operon expression

**DOI:** 10.1099/mgen.0.001019

**Published:** 2023-05-26

**Authors:** Aya Narunsky, Kumari Kavita, Shanker S. S. Panchapakesan, Megan E. Fris, Ronald R. Breaker

**Affiliations:** ^1^​ Department of Molecular, Cellular and Developmental Biology, Yale University, New Haven, CT 06511, USA; ^2^​ Howard Hughes Medical Institute, Yale University, New Haven, CT 06511, USA; ^3^​ Department of Molecular Biophysics and Biochemistry, Yale University, New Haven, CT 06511, USA; ^‡^​ Present address: Abcam, Branford, CT 06405, USA

**Keywords:** attenuation, gene regulation, intergenic region, RNA motif

## Abstract

Computational methods can be used to identify putative structured noncoding RNAs (ncRNAs) in bacteria, which can then be validated using various biochemical and genetic approaches. In a search for ncRNAs in *Corynebacterium pseudotuberculosis *, we observed a conserved region called the *ilvB*-II motif located upstream of the *ilvB* gene that is also present in other members of this genus. This gene codes for an enzyme involved in the production of branched-chain amino acids (BCAAs). The *ilvB* gene in some bacteria is regulated by members of a ppGpp-sensing riboswitch class, but previous and current data suggest that the *ilvB*-II motif regulates expression by a transcription attenuation mechanism involving protein translation from an upstream open reading frame (uORF or leader peptide). All representatives of this RNA motif carry a start codon positioned in-frame with a nearby stop codon, and the peptides resulting from translation of this uORF are enriched for BCAAs, suggesting that expression of the *ilvB* gene in the host cells is controlled by attenuation. Furthermore, recently discovered RNA motifs also associated with *ilvB* genes in other bacterial species appear to carry distinct uORFs, suggesting that transcription attenuation by uORF translation is a common mechanism for regulating *ilvB* genes.

## Introduction

Acetohydroxyacid synthase I (AHAS) and isomeroreductase (IR) are two key enzymes for the biosynthesis of the three branched-chain amino acids (BCAAs). These enzymes are encoded by the *ilvBNC* operon (wherein ‘*ilv’* represents isoleucine, leucine and valine). Specifically, AHAS is encoded by *ilvBN* and IR is encoded by *ilvC* [1, 2]. Although BCAAs are not synthesized by mammals, genes involved in their synthesis are vital for cell growth in many bacteria [3]. The phylogenetic distribution of these genes makes them potential targets for novel antibiotics, and thus the regulation of their expression has been studied extensively.

Computational methods offer powerful approaches for finding new structured RNA motifs, including those involved in gene regulation [4, 5]. For example, these methods were proven useful for identifying riboswitch candidates in bacteria [6–9]. Riboswitches are structured noncoding RNA (ncRNA) domains that regulate the expression of adjacent genes by forming a selective ligand-binding pocket or ‘aptamer’ [10–12]. As part of a search for structured ncRNAs in bacterial genomes, we observed an aptamer candidate upstream of the *ilvB* gene in *Corynebacterium pseudotuberculosis *. Previously, representatives of a ppGpp riboswitch class were reported to control this gene in Firmicutes [13]. Therefore, we hypothesized that the newly recognized RNA structure called the *ilvB*-II motif might represent a different riboswitch class selective for ppGpp. Genetic reporter assays conducted in a surrogate organism with a representative RNA derived from *Corynebacterium ulcerans,* an emerging human pathogen that causes respiratory diphtheria [14], indicate that the motif indeed regulates the expression of its downstream gene. However, experimental data also suggest that the RNA motif does not function as a ppGpp riboswitch, which prompted further examination of the mechanism of *ilvB* gene regulation in *Corynebacterium *.

Previous studies have shown that in *Escherichia coli* and in *Corynebacterium glutamicum *, an upstream open reading frame (uORF) associated with the *ilvBNC* operon controls its expression through an attenuation mechanism involving terminator and antiterminator sequences [15–17]. Different speeds of translation of the leader peptide encoded by the uORF result in changes to the levels of *ilvB* gene expression, often by affecting the formation of terminator and antiterminator stems. This precedent is consistent with features we observe in the motif identified in the present study. Specifically, the *ilvB*-II motif includes a conserved start codon always in-frame with a stop codon, and its conservation pattern is consistent with a protein coding region. Furthermore, we show that previously reported RNA motifs associated with *ilvB* genes in various bacterial species might also carry uORFs. These findings support the hypothesis that attenuation mechanisms are widely used for bacterial *ilvB* gene regulation.

## Methods

### Computational search for novel RNA motifs in *Corynebacterium*

The *ilvB*-II motif was detected as part of an effort to identify and classify functional ncRNAs in a collection of 50 bacterial genomes (Breaker Laboratory, unpublished data). Specifically, the motif was identified while searching the *C. pseudotuberculosis* genome [National Center for Biotechnology Information (NCBI) reference sequence: NC_017945.2]. The computational search pipeline [8, 9] is briefly described as follows. For each genome examined, all the intergenic regions (IGRs) that are long and enriched in G and C nucleotides relative to the average for the species were first identified. IGRs that met the length and GC content criteria were used as queries for Infernal [18]. Infernal enables the identification of additional IGRs that share sequence and structure similarities with query alignments, and was applied for the analysis of Reference Sequence (RefSeq) database release 80 and of metagenomic datasets [6, 19].

After an initial set of representatives was identified, CMfinder [20] was used to create a 2D structural model for the putative structured RNA class, based on covariance analysis. The Infernal search was then repeated with the resulting structural model to identify additional representatives. This collection of sequences is referred to as a ‘motif’, and the BLISS server (Breaker Laboratory Intergenic Sequence Server) [21] is used to aid in evaluating the annotations of genes in the same genomic neighbourhood. The motif is assigned a putative function based on various factors, such as its sequence, structure, orientation relative to surrounding genes and the annotations of the functions of surrounding genes. Finally, the R2R software program [22] is used to depict a consensus sequence and secondary structure model, highlighting covariation and conservation patterns.

### In-line probing assays

In-line probing assays [23] were used to evaluate the structural model and to assess direct binding of ligands using a described previously protocol [24]. The DNA oligonucleotides used to prepare (via PCR amplification) the double-stranded DNA template for *in vitro* transcription of the RNA construct are as follows: *ilvB-103F* 5′-TAATACGACTCACTATAggAACATTATTCGACTTGTAGTGCTATCCGAGCGGCACCTGCCGTAACGGCCACCA; *ilvB-103R* 5′-GCCCCCGATCAGCACTTGTGATGCTGGCGAGGGCGCTTACGTTACGACTTGGTGGCCGTTACGGCAGGTGCCGC.

### Genetic constructs and bacterial cells

DNA constructs used for *in vitro* transcription or for cloning of *ilvB*-II motif RNAs based on the genome of *C. ulcerans* 809 were supplied by Integrated DNA Technologies (Coralville, IA, USA). DNAs were amplified using PCR, and reporter gene constructs were cloned between a constitutive *Bacillus subtilis lysC* promoter and the *E. coli lacZ* gene on plasmid pDG1661 to create a transcriptional fusion. Plasmids carrying the *ilvB*-II motif reporter fusion constructs were subsequently transformed into *B. subtilis* 1A1 cells and integrated into the bacterial chromosome. Selection for proper chromosomal integration was achieved by using 5 µg ml^−1^ of chloramphenicol. Counter selection was accomplished using 100 µg ml^−1^ spectinomycin.

### Genetic reporter assays

Reporter assays were performed in liquid culture by inoculating *B. subtilis* in 2 ml of either lysogeny broth (LB) or Spizizen glucose minimal medium (GMM) [25], supplemented with 5 µg ml^−1^ chloramphenicol. The cultures were incubated overnight at 37 °C and then diluted 1/100 in LB or 1/10 in GMM with appropriate supplements [chloramphenicol at 5 µg ml^−1^ and X-gal (5-bromo-4-chloro-3-indolyl-β-d-galactopyranoside) at 100 µg ml^−1^]. The resulting mixtures were incubated at 37 °C for 24 h and images were recorded. Experiments were performed in triplicate, and representative data are depicted herein.

### Computational analysis of the uORF protein sequence

The uORF peptide sequence was translated using the Transeq tool [26]. The MAFFT sequence aligner was used to create a multiple sequence alignment of the peptides [27], and the WebLogo v3.7.4 tool was used to create a visual figure representing this alignment [28].

## Results and discussion

### Identifying the *ilvB*-II motif in *Corynebacterium*

**Fig. 1.**
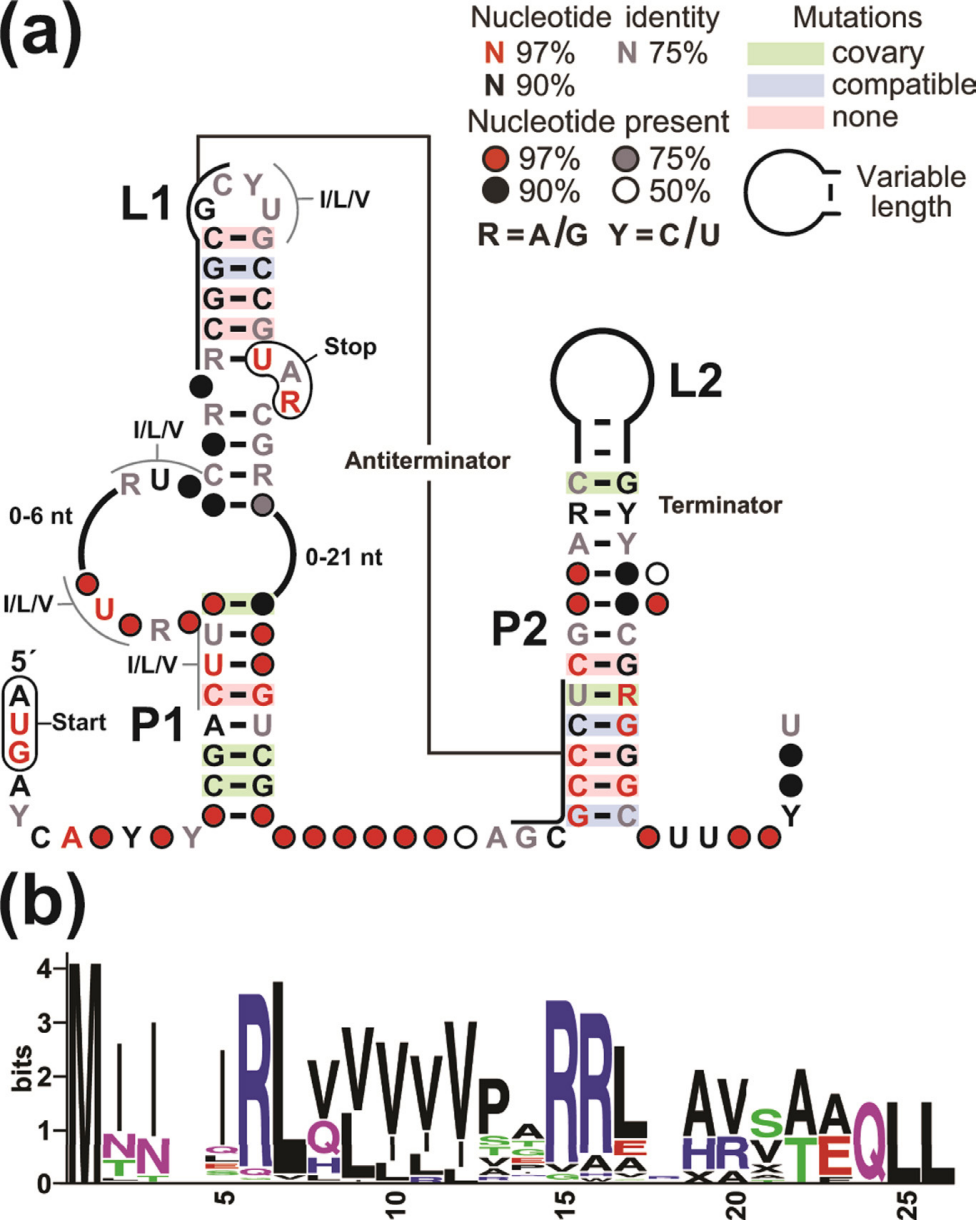
A conserved RNA sequence and structure upstream of *ilvB* genes in the genus *Corynebacterium *. (**a**) Consensus sequence and a secondary structure model of the *ilvB*-II motif, based on 103 representatives from various species of *Corynebacterium *. I/L/V identifies nucleotide triplets coding for isoleucine, leucine and valine amino acids. The denoted start and stop codons identify a predicted uORF. Predicted terminator and antiterminator base-paired substructures are also identified. (**b**) Consensus amino acid sequence of the short peptide encoded by the predicted *ilvB* uORF, as derived from the representatives identified in *Corynebacterium* (File S1) and generated by WebLogo [24]. Although the start codon was AUG, GUG and UUG, we set the first amino acid of the peptide to methionine.

We identified 103 unique sequence representatives in the genus *Corynebacterium* that conform to a specific sequence and secondary structure pattern we have named the *ilvB*-II motif due to their consistent location in the 5′ untranslated region (UTR) of an *ilvB* gene (File S1, available in the online version of this article). The structural model corresponding to the alignment includes two predicted stem loops called P1 (pairing element 1) and P2 (Fig. 1a). Both P1 and P2 carry several conserved nucleotides and exhibit a pattern of covariation in some locations. P2 is followed by a stretch of uridine nucleotides, which matches the arrangement observed for most intrinsic transcription terminator stems [29]. We also noted that the motif appears to include a uORF spanning most of the P1 region of the RNA (Fig. 1b). Previous bioinformatic searches also revealed that some *ilvB* genes are likely controlled by uORF-mediated transcription attenuation [30–32]. Nonetheless, because the motif is located upstream of a gene that in some bacteria is known to be regulated by a ppGpp riboswitch [13], we were motivated to test whether it represents a new riboswitch class for this nucleotide-like signalling molecule.

**Fig. 2.**
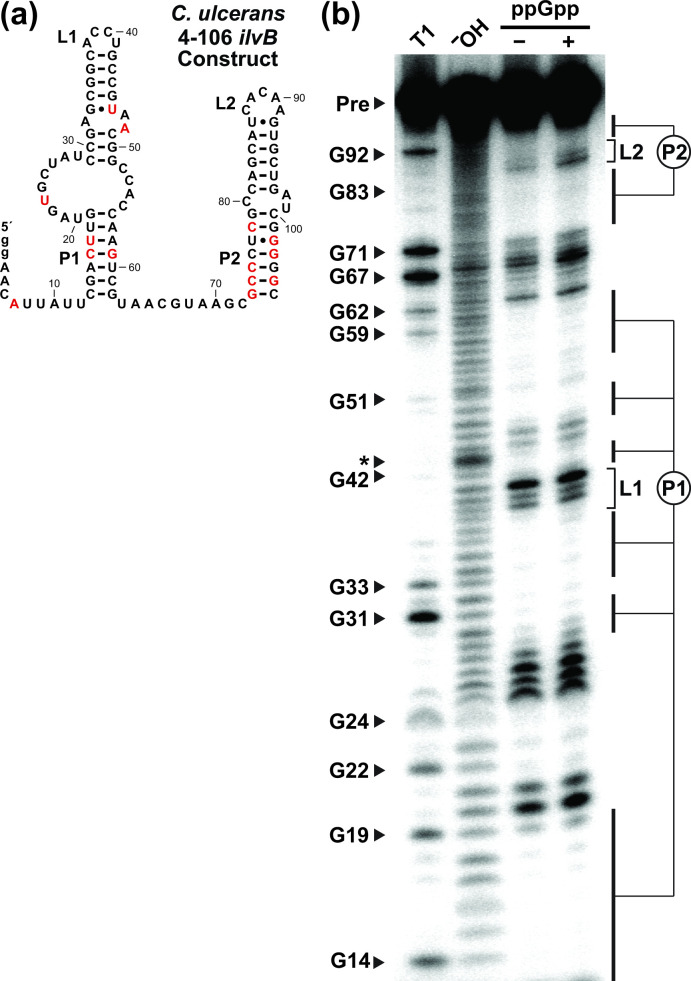
In-line probing of the *ilvB*-II motif from *C. ulcerans* confirms the bioinformatically predicted secondary structure. (**a**) Sequence and secondary structure model of the RNA construct subjected to in-line probing. The RNA sequence (called 4–106 *ilvB*-II) encompasses nucleotide positions 4 through 106 of the natural *C. ulcerans* sequence plus two G nucleotides (lowercase ‘g’ letters) added at the 5′ end to facilitate preparation by *in vitro* transcription. (**b**) Autoradiogram of the products of in-line probing reactions with the 5′ ^32^P-labelled 4–106 *ilvB*-II construct after separation by denaturing (8M urea) 10 % polyacrylamide gel electrophoresis (PAGE). T1 indicates the precursor RNA (Pre) was RNA subjected to partial degradation by treatment with RNase T1, which cleaves after G nucleotides (certain bands labelled). ^‒^OH indicates partial digestion of Pre RNA under alkaline conditions, which cleaves after all nucleotides. The asterisk identifies a compression site where several product bands migrate to the same location. In-line probing reactions were conducted either in the absence (‒) or presence (+) of 100 µM ppGpp. Bands corresponding to nucleotides involved in the predicted secondary (**p1 and p2**) structures are highlighted, and generally exhibit reduced spontaneous strand scission relative to regions in bulges, joining regions or loops (such as L1 and L2).

A structural probing assay called in-line probing [23, 24] was applied, which revealed that a representative *ilvB*-II motif RNA from *C. ulcerans* (Fig. 2a) indeed conforms to the predicted structure (Fig. 2b). However, the RNA does not exhibit structural modulation when ppGpp is introduced into the in-line probing reaction, suggesting that the RNA is unlikely to function as a riboswitch that directly senses ppGpp. Riboswitch aptamers commonly exhibit RNA folding differences in the absence versus presence of their target ligands [13]. Given the lack of evidence for ppGpp binding, we decided to investigate whether the motif regulates the expression of its downstream gene, perhaps by another mechanism.

### *In vivo* regulation of gene expression by an *ilvB*-II motif representative

**Fig. 3.**
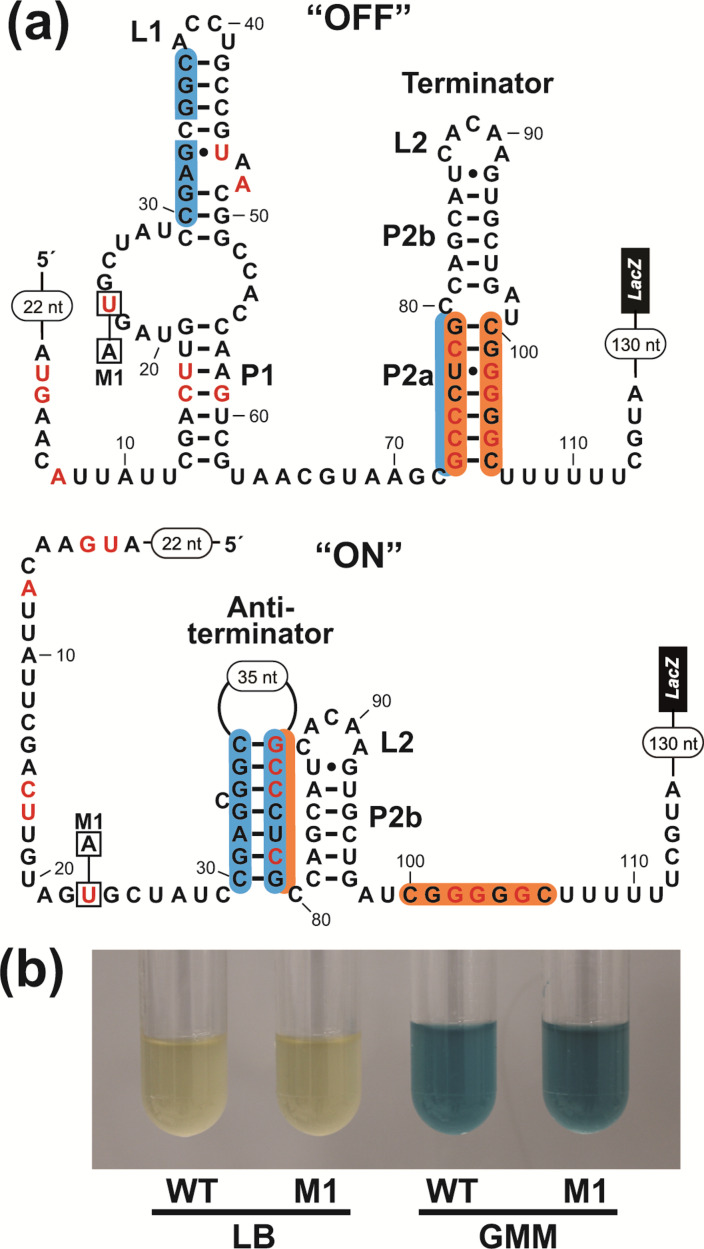
Genetic reporter fusion assays for the evaluation of an *ilvB*-II motif RNA representative. (**a**) Sequence and secondary structure models of the *C. ulcerans ilvB*-II motif representative prepared as a transcriptional fusion with the *E. coli lacZ* gene. Top: predicted structure of the RNA in the genetic ‘OFF’ state, wherein the lower part of stem P2 (denoted P2a; orange shading) participates in forming an intrinsic terminator stem. Bottom: predicted structure of the RNA in the genetic ‘ON’ state, wherein an antiterminator stem (blue shading) forms at the expense of the P2a stem. Red nucleotides identify positions in the consensus model (Fig. 1a) that are conserved in at least 97 % of the sequences present in the alignment. Encircled numbers represent natural sequences from the *C. ulcerans ilvB* gene that are not depicted. Construct M1 is a mutant version of the WT sequence that carries a U23A mutation. (**b**) *lacZ* reporter gene assays of the WT and M1 *ilvB*-II reporter constructs depicted in (**a**) cultured with rich (LB) or minimal (GMM) media. Blue colour indicates high *lacZ* expression.

We evaluated the gene control function of the representative *ilvB*-II motif RNA from *C. ulcerans* by transforming *B. subtilis* with a plasmid containing a transcriptional fusion of wild-type (WT) or mutant (M1) examples of the motif and a *lacZ* reporter gene (Fig. 3a). This construct has the potential to form two distinct structures corresponding either to the ‘OFF’ state that forms a terminator stem (Fig. 3a, top), or to the ‘ON’ state that forms the antiterminator structure (Fig. 3a, bottom). Specifically, the right shoulder of the lower portion of P2 (denoted P2a) (orange shading) can form an alternative base-pairing interaction with a region of the loop of P1 to form a putative antiterminator stem (blue shading).

Reporter strains carrying either the WT or M1 constructs were grown in either rich (LB) or minimal (GMM) media containing the β-galactosidase substrate X-gal, and reporter gene regulation was evaluated by visual inspection (Fig. 3b). The relative intensity of blue colour of the culture media is indicative of *lacZ* expression. With the WT construct, reporter gene expression was high in GMM in comparison to LB, revealing that the *ilvB*-II motif representative strongly suppresses reporter gene expression in rich media. This finding is consistent with the motif functioning as a genetic OFF switch, wherein the signalling molecule is abundant in rich media. Furthermore, this conclusion matches that reported previously for the regulation of *ilvB* gene expression [13, 16].

The M1 construct, which carries a mutation in a strictly conserved nucleotide in the consensus model (Fig. 3a) (U23A) exhibits no difference in the levels of gene expression (Fig. 3b), suggesting that this nucleotide is not essential for the regulatory effect. A conserved nucleotide in a riboswitch candidate is usually indicative of a functionally important nucleotide, and therefore a mutation at this position would be expected to disrupt riboswitch activity. If the motif functioned as a riboswitch that turns off expression when its ligand is present, a mutation that disrupted the aptamer would likely activate expression in rich media compared to the WT sequence. Given these results, we considered other hypotheses regarding the mechanism of gene control that do not involve riboswitch function.

### The *ilvB*-II motif exhibits the characteristics of a uORF

Previous studies in various bacterial species revealed that some *ilvB* genes are regulated either by a riboswitch or by a uORF [13, 16]. Riboswitches for ppGpp frequently regulate more than a single mRNA within the genomes of species that use them [13], whereas the RNA motif we identified is always found in a consistent location and only once in each genome. Representatives are typically located ~150 nucleotides upstream of the start codon for the ORF of the *ilvB* gene. Previous research revealed that *ilvB* in *C. glutamicum* is regulated by a uORF [16]. Importantly, our alignment of *ilvB*-II motif sequences (File S1) includes a representative from a strain of this same species (NCBI reference sequence: NC_009342.1). This observation reveals that this previously validated uORF matches the consensus for the *ilvB*-II motif identified in the current study (Fig. 1a).

As noted above, we recognized that a conserved start codon (AUG/GUG) resides at the beginning of the motif (Fig. 1a), always positioned in-frame with a downstream stop codon. The distance between the beginning of the start codon and the beginning of the associated stop codons is usually 45, 48, or 51 nucleotides, which defines a uORF coding for a peptide of 15–17 amino acids (Fig. 1b). The previously reported uORF is 45 nucleotides in length [16], which conforms to the consensus we have developed. However, some representative uORFs are as short as 30 nucleotides or as long as 72 nucleotides (excluding the stop codon) (File S1), but all lengths are divisible by three, suggesting codon function.

The consensus sequence also is enriched in codons for the three BCAAs, which are primarily located in four positions (Fig. 1a, I/L/V annotations). The M1 construct examined in our reporter gene assays alters the second of these codons by changing a valine codon to a glutamate codon. However, there are other I/L/V codons in the sequence, which might explain why the mutation does not disrupt gene regulation.

### Additional RNA motifs associated with *ilvB* genes suggest broader use of attenuation mechanisms

**Fig. 4.**
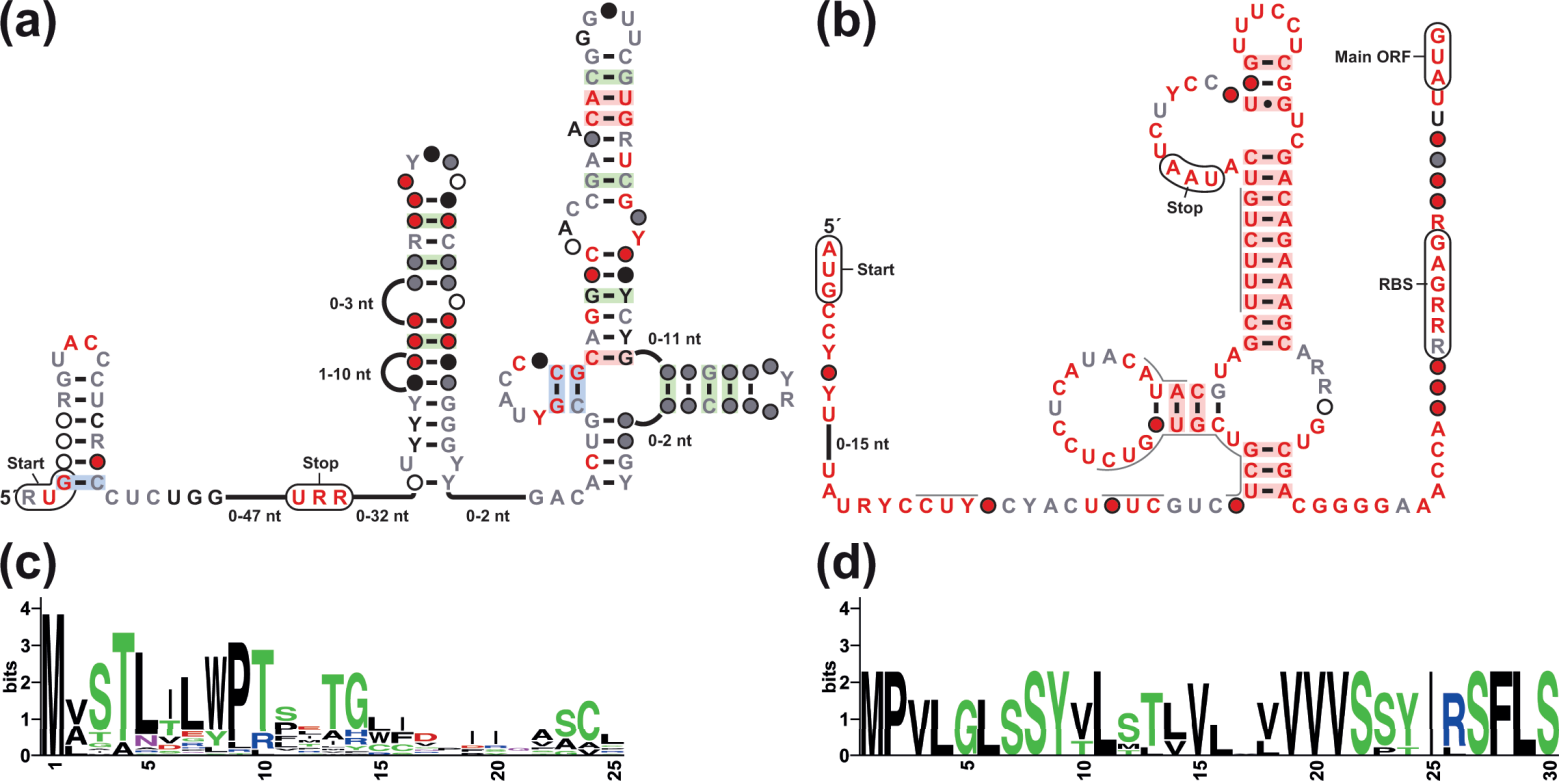
Previously identified RNA motifs associated with bacterial *ilvB* genes. (**a**) Consensus sequence and structural model for the *ilvB*-OMG motif updated from a model reported previously [6] (File S2). Annotations are as described for Fig. 1(a). (**b**) Consensus sequence and structural model for *ilvB* motif updated from a model reported previously [9]. (**c**) Consensus amino acid sequence of the short peptide encoded by the uORF predicted to be present in the *ilvB*-OMG motif [6] (File S3). Details are as described for Fig. 1(b). The height of each letter in the figure represents its frequency, corrected to the number of peptide sequences in the alignment. (**d**) Consensus amino acid sequence of the short peptide encoded by the uORF predicted to be present in the *ilvB* motif [9]. There are only four unique peptide sequences, and the height of each letter in the figure is corrected accordingly.

Two additional RNA motifs associated with *ilvB* genes from other bacterial lineages have been reported by our laboratory previously [6, 9]. Based on the rarity and the lack of clues regarding the possible functions of these motifs, our hypotheses regarding possible functions were uncertain. Specifically, the first RNA structure, called the *ilvB*-OMG motif (Fig. 4a; File S2), was noted as an RNA of unknown function [6]. The second structure, simply called the *ilvB* motif (Fig. 4b; File S3), was considered a weak riboswitch candidate [9], meaning that there was low confidence in the riboswitch hypothesis. Thus, we had prioritized other candidate riboswitch classes for experimental investigation over the analysis of these motifs.

Considering the findings described herein with the *Corynebacterium ilvB*-II motif, we reexamined the sequence alignments and features of the *ilvB*-OMG and *ilvB* motifs to determine whether the uORF mechanism could be more broadly distributed than originally known. The *ilvB*-OMG motif [6] consensus sequence and structural model was updated based on 41 representatives from various bacterial phyla. All but two carry a start codon in-frame with a downstream stop codon, suggesting that these sequences might function as uORFs. Three other representatives were much longer than others, and were removed from the protein alignment despite carrying a similar amino acid consensus sequence. Although the peptides that would result from translation of these predicted uORFs are highly variable in length and sequence, they are rich in codons for BCAAs and threonine (Fig. 4c). These features of *ilvB*-OMG motif RNAs are suggestive of attenuation function in response to the availability of aminoacylated BCAA tRNAs.

The *ilvB* motif [9] only has seven representatives present in species of *Leptospira *. After updating the consensus sequence and structural model (Fig. 4b), this motif appears to carry a start codon in-frame with a downstream stop codon, located ~70 nucleotides upstream of the main ORF. The RNA sequence exhibits a conservation pattern consistent with a uORF region that is also rich in BCAAs and in threonine (Fig. 4d). For example, all representatives have at least three consecutive valine codons. Again, this architecture is consistent with an attenuation function, although confirmation of the functions of the *ilvB*-OMG and *ilvB* motifs requires additional genetic experimentation.

## Conclusion

Previous studies [15–17] have demonstrated that the leader sequence of the *ilvB* gene can control the expression levels of *ilvB* by exploiting a uORF regulating the formation of an intrinsic terminator stem [29, 33]. Likewise, we observe a conserved uORF associated with a strong terminator stem (pairing element P2) in the leader sequences of the *ilvB* mRNAs of many *Corynebacterium* species. Furthermore, when fused to a *lacZ* reporter gene, the *ilvB*-II motif from the mRNA leader sequence of *C. ulcerans* exhibits high expression levels in minimal media and low levels in rich media (Fig. 3b).

These observations suggest that the motif representative from this species also regulates the expression of its associated main ORF by encoding a short peptide enriched in BCAAs, which can only be translated quickly when sufficient concentrations of isoleucine, leucine and valine are present in the media. Thus, we speculate that the secondary structure features characteristic of the *ilvB* motif are differentially formed based on the speed of translation of the uORF, which would cause transcription termination if the P2 stem (intrinsic terminator hairpin) is formed. This proposed uORF-mediated gene regulation mechanism for *ilvB* resembles the attenuation mechanism originally reported for the regulation of tryptophan biosynthesis genes [33]. In the abundance of tryptophan, a uORF is translated quickly, preventing the formation of an antiterminator hairpin. This, in turn, allows a terminator stem to form, resulting in decreased levels of RNA transcription of the adjacent gene.

Most known classes of riboswitches are widespread among diverse lineages of bacteria [12, 34]. However, the riboswitch classes discovered more recently tend to be rarer and more narrowly distributed phylogenetically. Therefore, structured RNA motifs that are only present in a few closely related species might be less likely to function as metabolite-binding riboswitches, but rather serve as gene control devices that are bound by protein-based genetic factors or as uORFs like those described herein. Regardless, we anticipate that many novel types of RNA-based regulatory systems remain to be discovered.

## Supplementary Data

Supplementary material 1
