# Supplementary material for: A conserved uORF in the ilvBNC mRNA of Corynebacterium species regulates ilv operon expression
Source: Microb Genom. 2023 May 26;9(5):mgen001019. doi: 10.1099/mgen.0.001019 (PMC10272879; doi:10.1099/mgen.0.001019)
Supplement: Supplementary material 1 [file mgen-9-1019-s001.pdf]

Supplementary Information

A conserved uORF in the *ilvBNC* mRNA of *Corynebacterium* species regulates *ilv* operon expression

Aya Narunsky[1]†, Kumari Kavita[1]†, Shanker S. S. Panchapakesan[1]†, Megan E. Fris[2,‡], and Ronald R. Breaker[1,2,3,*]

Author affiliations: [1]Department of Molecular, Cellular and Developmental Biology, Yale University, New Haven, CT 06511, USA; [2]Howard Hughes Medical Institute, Yale University, New Haven, CT 06511, USA; [3]Department of Molecular Biophysics and Biochemistry, Yale University, New Haven, CT 06511, USA

†These authors contributed equally to this work
‡Present address: Abcam, Branford, CT 06405
***Correspondence**: Ronald R. Breaker, ronald.breaker@yale.edu

File S1. Sequence alignment of the *ilvB*-II motif.

```
# STOCKHOLM 1.0
#=GF SORT_STATS 98 168 0
NZ_AFYA01000002.1/29005-28884
AUGACCAUU...AUUCGACUUGUAGUA......GUA..ACCG.CGCGGCGCCUGCCG.........................UAA.CGGC.................CUUCC.AA.
GUC.GUCUCGUC....AAGCGCCCUCGA...CAACAC..UCAC.CACA..GUGUUG...GAACGA..GGGCUUUCUUGUUG
NZ_KV812479.1/16371-16504
AUGAUCAUU...AUUCGACUUCACCUCGUAGUAGUA..CCCG.GGCGGCGCGAGCCG.........................UAG.CGAGUC............CACAAGUG.AC.
GUC.GACCAGCA.....AGCGCCCCCGU...UACCUU.CACAC..CCAGGAAGGAA...AGACGG..GGGCGUUAGUCGUU
NZ_KV806831.1/110751-110618
AUGAUCAUU...AUUCGACUUCACCUCGUAGUAGUA..CCCG.GGCGGCGCGAGCCG.........................UAG.CGAGUC............CACAAGUG.AC.
GUC.GACCAGCA.....AGCGCCCCCGU...UACCUU.CACAC..CCAGGAAGGAA...AGACGG..GGGCGUUACUCGUU
NZ_KV794854.1/187802-187670
AUGAUCAUU...AUUCGACUUCACCUCGUAGUAGUA..CCCG.GGCGGCGCGUGCCG.........................UAG.CGAGUC............CACUAGUG.AC.
GUC.GACCAGCA.....AGCGCCCCCGU...UACCUU.CACAC..CCAGGAAGGA....CAGCGG..GGGCGUUAUUUGUU
NZ_JIAJ01000008.1/123276-123400
AUGAUCAAC...AUUCGACUUGUGGUA......GUA..GCUG.CACGGCGCCUGCCG.........................UGA.CGGUCUG..............ACCUC.CA.
GUC.GUCUCGAC....AAGCGCCCUCGA...CAGCAC..CCACCCACA..GUGCUG....UUCGG..GGGCUUUGUCUUUU
NZ_ATVF01000002.1/139928-139807
AUGACCAUU...AUUCGACUUGUACUA......GUA..ACCC.CACGGCGCCUGCCG.........................UAG.CGGC..................CUACCAA.
GUC.GUCUCGUC....AAGCGCCCUCGA...CAACAC..UCAC.CACA..GUGUUG...GAACGA..GGGCUUUCUUGUUG
NZ_JUMN01000085.1/2198-2330
AUGAUCAUU...AUUCGACUUCACCUCGUAGUAGUA..CCCG.GGCGGCGCGAGCCG.........................UAG.CGAGUC............CACUAGUG.AC.
GUC.GACCAGCA.....AGCGCCCCCGU...UACCUU.CACAC..CCAGGAAGGA....CAGCGG..GGGCGUUAUUAGUU
NZ_ACLH01000005.1/107419-107286
AUGAUCAUU...AUUCGACUUCACCUCGUAGUAGUA..CCCG.GGCGGCGCGAGCCG.........................UAG.CGAGUC............CGCUAGUG.AC.
GUC.GACCAGCA.....UGCGCCCCCGU...UACCUU.CACAC..CCAGGAAGGAA...AGACGG..GGGCGUUAUUCGUU
NZ_BCRC01000002.1/59853-59980
AUGAUCAUU...AUUCGACUUCACCUCGUA...GUA..CCCG.GGCGGCGCGAGCCG.........................UAA.CGAGU.............CCACAGUG.AC.
GUC.GACCUGC......AGCGCCCCCGC...UACCUU.CACAC..CCA.GAAGGAC...AGACGG..GGGCGUUAGUUAUA
NZ_JRUB01000003.1/93397-93266
AUGAUCAUU...AUUCGACUUCAGCUUCCGGUAGUA..GUAG.CGCGGCGCUUGCCG.........................UAG.CGACAC............CCCAGUUG.UU.
GUC.GAUAUUGC....AAGCGCCCUCGC..UCAGCUU..CUAC..C.C..AAGCUG...AGACGG..GGGCUUUGCUAUAU
NZ_JVTN01000036.1/102026-102165
AUGAUCAUU...AUUCGACUUCAGCUAGUA...GUA..CCGG.CGCGGCGUUUGCCG.........................UAA.CGGGUUGCCGAAUCAAACUU.CAGCG.AC.
GUC.GAAAAGCA.....AGCGCCCUCGC...CAACUU.CUUAG..CGG..AAGUUG...AGACGG..GGGCGUUGGCAUUU
```

```
NZ_KQ959770.1/30883-31013
AUGAUCAUU...AUUCGACUUCAGCUUGUA...GUA..CCGGACGCGGCGCCUGCCGCAA.CGGCA................UAA.......................GUUG.UC.
GUU.GAUUAGAG...CAAGCGCCCUCGC...CAGUUG.CACAC..ACC.GCAACUG...AGACGG..GGGCUUUGUUGUUG
NZ_LTEB01000025.1/84190-84331
AUGAUCAUU...AUUCGACUUCAGCUAGUA...GUA..CCGG.CGCGGCGUUUGCCGCAU.CGAGUCGCCGAGCAACUUCUUUAG.CG.........................AC.
GUC.GAAAAGCA.....AACGCCCUCGC...CAACUA.CCUAC..C.AGGCAGUUG...GAACGA..GGGCUUUUGUUAUU
NZ_KV805153.1/141775-141907
AUGAUCAUU...AUUCGACUUCACCUCGUAGUAGUA..CCCG.GGCGGCGCGAGCCG.........................UAG.CGAAUC............CACUAGUG.AC.
GUC.GACCAGCA.....AGCGCCCCCGU...UACCUU.CACAC..CCAGGAAGGA....CAGCGG..GGGCGUUAUUAGUU
NZ_KV825630.1/100654-100524
AUGAUCAAC...CAGCGACUAGUAGUACGA...AUU..CCCA.CGCGGCGCUUGCCG.........................UAG.CGGUU.............CGUUUUUC.UAC
GCC.GCUGAAAC.AACAAGCGCCCUCGA...CAGCAC..GCAC....C..GUGCUG...AUUCGG..GGGCUUUUGUUUUG
NZ_ACSH02000008.1/90454-90326
AUGAACAUU...AUUCAGCUUGUACUCGUG...GUA..AUUGUCGCGGCGCCUGCCGUGU.CAACUGCC.............UAG........................UGC.AA.
GUU.GAUUUGUC....AAGCGCCCUCGG..UUCACGC...CAC..A.A..GUGUUG...AAGCGA..GGGUUUUUGCAUAG
NZ_CP010827.1/1294698-1294827
AUGAUCAUU...AUUCGACUUCACCUCGUA...GUA..CCCG.GGCGGCGCGAGCCG.........................UAA.CGAGU.............CCACAGUG.AC.
GUU.GACCUACA.....CGCGCCCCCGC...UACCUU.CACACCCACG..AAGGAC...AGACGG..GGGCGUUAGUCGUA
NZ_LGSX01000012.1/4538-4653
AUGAACAUU...AUUCGACUUGUAGUG......CUA..UCCG.AGCGGCGCCUGCCG.........................UAA.CGG..................CCACC.AA.
GUC.GUAACGUA.....AGCGCCCUCGC...CAGCAU...CAC..A.A..GUGCUG....AUCGG..GGGCUUUUUUCGUA
NZ_CP011095.1/996452-996567
AUGAACAUU...AUUCGACUUGUAGUG......CUA..UCCG.AGCGGCGCCUGCCG.........................UAA.CGG..................CCACC.AA.
GUC.GUAACGUA.....AGCGCCCUCGC...CAGCAU...CAC..A.A..GUGCUG....AUCGG..GGGCUUUUUUUCGU
SRS018157_Baylor_scaffold_43439/431-303
AUGAACAUU...AUUCAGCUUGUACUCGUG...GUA..AUUGUCGCGGCGCCUGCCGUGU.CAACUGCC.............UAG........................UGU.AA.
GUU.GAUUUGUC....AAGCGCCCUCGG..UUCACGC...CAC..A.A..GUGUUG...AAGCGA..GGGUUUUUGCAUAG
NZ_LDYD01000008.1/204736-204866
AUGAUCAUU...UUGCGACUAGUAGUG......GUA..CCGC.GACGGCGCCUGCCG.........................UGA.CGGUGAG.........AAACCACUUC.UC.
GUC.GUUGUGAC....AAGCGCCCUCGA...CAGCAC..CCACCCACA..GUGCCU...GUACGA..GGGCUUUGUUAUGU
NZ_LXGG01000012.1/107049-106925
GUGAUCAAC...GAGCGACUUGUAAUUAUU...CUC..UCCC.GACGGCGCUUGCCG.........................UAG.CGGUG.............CGUUUUAC.AC.
GCC.GCUCACAC.ACCAAGCGCCCUCGA...GAGC......AC..AUC....GCUC....GUCGG..GGGCUUUUGUUGUA
SRS015044_WUGC_scaffold_38868/315-195
AUGACCAUU...AUUCGACUUGUACUC......GUA..CGCC.CUCGGCGCUUGCCG.........................UAA.CGGU.................UAUAC.AA.
GUC.GUUUCGUC.....AGCGCCCUCGG...AAGCAC.CACAC.CACACGGUGC......UUCGG..GGGCUUUGAUUUUU
NZ_BCSU01000002.1/86538-86657
AUGAUCACC...AUUCGACUUGUG.........GUA..GCAG.CGCGGCGCCUGCCG.........................UAA.CGACC..................UACCCA.
GUC.GUCUCGAC....AAGCGCCCUCGA...CAGCAC..CCACCAUCA..GUGCUG....UACGG..GGGCUUUGUCUUUA
```

```
NZ_JPJB01000001.1/90561-90446
GUGAACAUU...AUUCGACUUGUAGUG......CUA..CCCG.AGCGGCGCCUGCCG.........................UAG.CGGC..................CAUC.AA.
GUC.GUAACGUA.....AGCGCCCUCGC...CAGCAU...CAU..A.A..GUGCUG....AUCGG..GGGCUUUUUUCGUA
NZ_CP013699.1/1904309-1904194
GUGAACAUU...AUUCGACUUGUAGUG......CUA..CCCG.AGCGGCGCCUGCCG.........................UAG.CGGC..................CAUC.AA.
GUC.GUAACGUA.....AGCGCCCUCGC...CAGCAU...CAU..A.A..GUGCUG....AUCGG..GGGCUUUUUCGUAG
NZ_KV788396.1/85810-85934
GUGAUCAAC...GAGCGACUUGUAAUUAUU...CUC..UCCC.GACGGCGCUUGCCG.........................UAG.CGGGUG............CGUUUUAC.AC.
GCC.GCUGACAC.ACCAAGCGCCCUCGA...GAGC......AC..GAC....GCUC....GUCGG..GGGCUUUUGUUGUA
NZ_JAQQ01000012.1/73281-73397
AUGAACAUU...AUUCGACUUGUAGUG......AUU..ACCA.CUCGGCGCUUGCCG.........................UAG.CGG...................CUAC.AA.
GUC.GUUUCCGA...AAAGCGCCCUCGA...CAGCA...CCAC..ACA...UGCUG....AGCGG..GGGCUUUCCUUAUA
NZ_CP012342.1/904626-904498
AUGAUCACG...ACGCGACUUGUAGUAGUA...GUAAUUCCGAUGCGGCGCUUGCCG.........................UAAGCGGGUUGC.........ACUCAAAGC.UA.
GCC.GCAAACCA.....AGCGCCCUCGA...GAGCUUC............UAGCUC...GCUCGA..GGGCUUUUUGUUUG
NZ_AQUX01000009.1/35144-35016
AUGAUCAUU...GAACGGCUACAGGUA......AUU..AUUC.CGCGGCGCUUGCCG.........................UAG.AAGACG............AUUACCUGAUC.
GUC.GUUUAGGA...CAAGCGCCCUCCU...CAGCAC..CCACCCA..UGGUGCUG....GUAGA..GGGCGCUUGUCAUG
NZ_MLCQ01000018.1/86330-86458
AUGAUCAUU...UCGCGCGUACUAGUCAUU...CUUAUUCCG.CCCGGCGCUUGCCG.........................UAG.CGGACUGC..........UUGCCUAG.ACA
GUC.GCGAACUC....AAGCGCCCUCGA...CAGC.....CAC..ACG....GCUU...GGUCGG..GGGCUUUUGUUUUA
NZ_CP017639.1/1290196-1290067
AUGAUCAUU...UCGCACGUACUAGUCAUU...CUUAUUCCGCACCGGCGCUUGCCG.........................UAG.CGGACUGC..........UUGCCUAG.UC.
AGUCGCGAACUC....AAGCGCCCUCGA...CAGC.....CAC..ACG....GCUU...GGUCGG..GGGCUUUUGUUUUA
NZ_JVVY01000009.1/43665-43534
GUGAUCAACGAGAUUCGACUUCAAGUAGUA.................GUGC...CC..........................UAGGGGGCGUGCCCGAACGGGACACACUUG.CA.
GCC.GUAUUAAC....GCACGCCUCCGA...CAGCAC..CCAC.CACA..GUGCAC..AUGUCGG..GGGCUUUUGUUGUG
NZ_JIAH01000006.1/288993-289124
GUGAUCAACGAAAUUCGACUUCAAGUAGUA.................GUGC...CC..........................UAGGGGGCGUGCCCGAACGGGACACACUUG.CA.
GCC.GCAUUAAC....GCACGCCUCCGA...CAGCAC..CCAC.CACA..GUGCGA..AUGUCGG..GGGCUUUUGUUGUG
NZ_KI515742.1/108057-107949
AUGAUCAUU...AUUCGACUUCAGGUG......GUA..CGAGCGCGGCGG................................UGACCGCGA.CGUU...........UCCUG.AAC
GUC.GACGCUUU.......CGCCCCCGC...AGAC.....CAC..C.G....GC.....CCGCGG..GGGCUUUAG..UUG
NZ_GL397138.1/1172545-1172653
AUGAUCAUU...AUUCGACUUCAGGUG......GUG..CGAGCGCGGCGG................................UGACCGCGG.CGUU...........UCCUG.AAC
GUC.GACGCUUU.......CGCCCCCGC...AGAC.....CAC..C.G....GC.....CCGCGG..GGGCUUUAG..UUG
NZ_AUAQ01000016.1/35487-35612
AUGACGAAC...CUUCGACUUGUGAUU..A...UUA..GCCA..CCGGCGCUUGCCG.........................UAG.CGGCUA............UUAUUG...UC.
GUC.GAACGUCA.....AGCGCCCUCGG...CGGACA.CCCAU.CACG.GUGUUCG...UACCGA..GGGCUUCGGUGUUU
```

NZ_LZMN01000041.1/68611-68480
GUGAUCAACGAAAUUCGACUUCAAGUAGUA.................GUGC...CC..........................UAGGGGGCGUGCCCGAACGGGACACACUUG.CA.
GCC.GCAUUAAC....GCACGCCUCCGA...CAGCAC..CCAC.CAUA..GUGCGA..AUGUCGG..GGGCUUUUGUUGUG
NZ_CP011546.1/989572-989702
AUGAUCAU......CGAGCUAAUUCUUGUA...GUA..CCCA.CUCGGCGCUUGCCG.........................UAG.GGGCGAC..........UACCUGAAC.UC.
GCU.CACCUCGA...UAAGCGCCCUCGC...CAGCAC..CCACCCUUA..GUGCUG...AGACGA..GGGCAUUGUCAUUA
NZ_KB903378.1/71169-71044
AUGGACAUU...AUUCGACUUGUACUC......GUG..CACC.CGCGGCGCUCGCCG.........................UGA.CGGCCU...............GAUAC.AA.
GUC.GUUGCAGC....GAACGCCCUCAC...CGGGAG.CUAAC..CCAAAGCCUCG...GUAGCA..GGGCGUUUUUUCUG
NZ_KV823458.1/28976-28842
AUGAUCAUU...AUUCGACUUCAGCUAGUACUCGUA..CCCG.AGCGGCGCUCGCCG.........................UAG.CGGCU................AACUG.UU.
GUC.GUAUACGU...CAAGCGCCCUCGACGCGGAACC.ACCAG..AGGCAGGUUCC...CGCCGG..GGGCUUUGUCGUAU
NZ_CP008944.1/1338751-1338620
AUGGACAUU...AUUCGACUUGUACUC......GUA..UCCC.GGCGGCGCCUGCCG.........................UAG.GACG.................AUUCC.AA.
GUC.GAUUCCAGG..CAAGCGCCCACGG..AAAGCCA.GCCGC..ACUAGCGGCCAGCACUCCGGACGGGCGCUUUUCUUU
NZ_FJVG01000020.1/152923-152795
AUGAUCAAC...ACGCGACUUGCUGUAGUAC.U..UAUUCCGAUGCGGCGCUUGCCG.........................UAG.CGGUCAG.........UCACAUCUGU.AA.
GCC.GCCCAAAC...CAAGCGCCCUCGA...GAGC.......U..UCG....GCUC...GCUCGG..GGGCUUUUGUUUUG
NZ_KI515749.1/1231455-1231563
AUGAUCAUU...AUUCGACUUCAGGUG......GUG..CGAG.CGCGGCGG...............................UGACCGCGGCGUU............UUCUG.AAC
GUC.GACGCUUU.......CGCCCCCGC...AGAC.....CAC..C.G...GC.....CCGCGG..GGGCUUUAGUU..G
SRS054061_LANL_scaffold_4713/219-329
AUGAUCAUU...AUUCGACUUCAGGUA......GUA..CCAA.CGCGGUGG...............................UGACCGCGGCGC.............CCAUG.AC.
GUC.GACGCUUU.....UCGCCCCCGC...AGAC......AU.AACA...GC.....CGGCGG..GGGCUUUAUUCAUA
NZ_ACVP01000012.1/205927-206038
AUGAUCAUU...AUUCGACUUCAGGCA......GUA..CCAA.CGCGGUGG...............................UGACCGCGACGCU............UCAUG.AC.
GUC.GACGCAUU.....UCGCCUCCGC...AGAC......AG.AAAA...GC.....CCGCGG..GGGCUUUGUUCAUA
NZ_JVXO01000045.1/2686-2576
AUGAUCAUU...AUUCGACUUCAGGUA......GUA..CCAA.UGCGGCGG...............................UGACCGCGGCGC.............UCAUG.AC.
GUC.GACGCUUU.....UCGCCCCCGC...AGAC....ACAC..C.A...GC.....CCGCGG..GGGCUUUGUUCAUA
NZ_JUOH01000021.1/24827-24716
AUGAUCAUU...AUUCGACUUCAGGUA......GUA..CCAA.CGCGGUGG...............................UGACCGCGACGCC............UCGUG.AC.
GUC.GACGCUUU.....UCGCCCCCGC...AGACAG....AC..A.A...GC.....CCGCGG..GGGCUUUGUUCAUA
NZ_KV786254.1/3984-3856
AUGAUCACG...AUGCGACUUGUAGUAGUA...GUAAUUCCGAUGCGGCGCUUGCCG.........................UAAGCGGGUUG.........CACUCAAAGC.UA.
GCC.GCAAACCA.....AGCGCCCUCGA...GAGCUU.........C..UAGCUC...GCUCGA..GGGCUUUUUGUUUG
NZ_HE998590.1/134951-134825
AUGACCAAU...UUUCGACUAGUGGUACUC...AUU..CCGG.UGCGGCGCUUGCCG.........................UAG.CGGCCUG..........AAUUCCUAC.UCA
GUC.GCCACAAC....AAGCGCCCCCGC..ACAGC......AC..UCA..C.GCUG....CCCGG..GGGCUUUGUUUUUG

```
NZ_KV810470.1/56721-56848
AUGAUCAUU...AUUCGACUUCACCUCGUA...GUA..CCCG.GGCGGCGCGAGCCG.........................UAA.CGAGUC............CA.CAGUG.AC.
GUC.GACCUGC......AGCGCCCCCGC...UUCCUU.CACAC..CCA.GAAGGAC...AGACGG..GGGCGUUAGUUAUA
NZ_KV811674.1/138915-138782
AUGAUCAUU...AUUCGACUUCACCUCGUAGUAGUA..CCCG.GGCGGCGCGAGCCG.........................UAG.CGAGUC............CGCUAGUG.AC.
GUC.GACCAGCA.....UGCGCCCCCGU...UGCCUU.CACAC..CCAGGAAGGAA...AGACGG..GGGCGUUAUUCGUU
NZ_KV813939.1/63684-63816
AUGAUCAUU...AUUCGACUUCCCCUCGUAGUAGUA..CCCG.GGCGGCGCGAGCCG.........................UAG.CGAGUC............CACUAGUG.AC.
GUC.GACCAGCA.....AGCGCCCCCGU...UACCUU.CACAC..CCAGGAAGGA....CAGCGG..GGGCGUUAUUAGUU
NZ_KV827677.1/109256-109124
AUGAUCAUU...AUUCGACUUCACCUCGUAGUAGUC..CCCG.GGCGGCGCGAGCCG.........................UAG.CGAGUC............CACUAGUG.AC.
GUC.GACCAGCA.....AGCGCCCCCGU...UACCUU.CACAC..CCAGGAAGGA....CAGCGG..GGGCGUUAUUAGUU
NZ_KV819530.1/119772-119904
AUGAUCAUU...AUUCGACUUCACCUCGUUGUAGUA..CCCG.GGCGGCGCGAGCCG.........................UAG.CGAGUC............CACUAGUG.AC.
GUC.GACCAGCA.....AGCGCCCCCGU...UACCUU.CACAC..CCAGGAAGGA....CAGCGG..GGGCGUUAUUAGUU
NZ_GG667531.1/201745-201884
AUGAUCAUU...AUUCGACUUCAGCUAGUA...GUA..CCGG.CGCGGCGUUUGCCG.........................UAA.CGGGUUGCCGA.AUCAAACUUCAGCG.AC.
GUC.GAAAAGCA.....AGCGCCCUCGU...CAACUU.CUUAG..CGG..AAGUUG...AGACGG..GGGCGUUGGCAUUU
NZ_CP014635.1/369767-369626
AUGAUCAUU...AUUCGACUUCAGCUAGUA...GUA..CCGG.CGCGGCGUUUGCCGCAU.CGAGUCGCCGAGCAACUUCUUUAG.........................CG.AC.
GUC.GAAAAGCA.....AACGCCCUCGU...CAACUA.GCUAC..CAG.GUAGUUG...GAACGG..GGGCUUUUGUUAUU
NZ_KV814065.1/363653-363794
AUGAUCAUU...AUUCGACUUCAGCUAGUA...GUA..CCGG.CGCGGCGUUUGCCGCAU.CGAGUCGCCGAGCAACUUCUUUAG.........................CG.AC.
GUC.GAAAAGCA.....AACGCCCUCGC...CAACUA.CCUAC..CAG.GUAGUUG...GAACGG..GGGCUUUUGUUAUU
NZ_CP014634.1/229072-228931
AUGAUCAUU...AUUCGACUUCAGCUAGUA...GUA..CCGG.CGCGGCGUUUGCCGCAU.CGAGUCGCCGAGCAACUUCUUUAG.........................CG.AC.
GUC.GAAAAGCA.....AACGCCCUCGU...CAACUA.CCUAC..CAG.GCAGUUG...GAACGA..GGGCUUUUGUUAUU
NZ_KV788547.1/128803-128944
AUGAUCAUU...AUUCGACUUCAGCUAGUA...GUA..CCGG.CGCGGCGUUUGUCGCAU.CGAGUCGCCGAGCAACUUCUUUAG.........................CG.AC.
GUC.GAAAAGCA.....AACGCCCUCGU...CAACUA.CCUAC..CAG.GCAGUUG...GAACGA..GGGCUUUUGUUAUU
NZ_GG771307.1/192410-192534
AUGAUCAUU...AUUCGACUUCAGGUGGUA...GUA..GCAG.CACGGCACUUGCCG.........................UAG.CGGC.................CGUUG.AA.
GUC.GUUGAAAAG..CAAGUGCCCUCGC...CAACUC..CCAC..C.C..GAGUUG...AGUCGG..GGGCAUUGUUCAUU
NZ_CAFW01000088.1/35810-35682
AUGAUCAUU...AUUCGACUUCAGGUGGUAGUAGUA..GCAG.CGCGGCACUUGCCG.........................UAG.CGGCC................GUUUG.AA.
GUC.GUUGAAUAG..CAAGUGCCCUCGC...CAACUC..CCAC..C.A..GAGUUG...AGUCGG..GGGCAUUGCUCAUU
NZ_LSTQ01000006.1/27986-27858
AUGAUCAAU...AUUCGACUUCAGGUGGUAGUAGUA..GCAG.CGCGGCGCUUGCCG.........................UAG.CGGCCA...............UUUUG.AA.
GUC.GUUAAAAG...CAAGUGCCCUCGC...CAACUC..CCAC..C.C..GAGUUG...AGUCGG..GGGCAUUGUUUAUU
```

NZ_CP014279.1/1680367-1680239
AUGAUCAAU...AUUCGACUUCAGGUGGUAGUAGUA..GCGG.CGCGGCGCUUGCCG.........................UAG.CGGCCA...............UUUUG.AA.
GUC.GUUAAAAG...CAAGUGCCCUCGC...CAACUC..CCAC..C.C..GAGUUG...AGUCGG..GGGCAUUGUUUAUU
NZ_AQUV01000003.1/83105-82975
AUGAUCACC...GCGCGACUAGUAGUAAUU...AUU..CCGU.CUCGGCGUUUGCCG.........................UAG.CGGUCUG..........AUUUCCUGC.UCA
GUC.GCCACAAC....AAACGCCCUCGA...CAGCGC..CGAA..CCG..GUGCCU....GUUGA..GGGUUUUUGUUUAG
NZ_GG667195.1/66867-66996
AUGAUCAAU...UCGGGACUAGUAGUAAUU...AUU..GCCGGUGCGGCGCUUGCCG.........................UAG.CGGUCUG..........AUCUUCUGC.UCA
GUC.CCUCAACA.....AGCGCCCUCGA...CAGCAC..CUGA....A..GUGCUG...GACCGA..GGGCAUUCGUUUUU
NZ_MLAL01000036.1/25124-25251
AUGAUCAUU...UCGCGCGUACUAGUCAUU..C.UUAUUCCG.CCCGGCGCUUGCCG.........................UAG.CGGACUG..........CUUGCCUAG.UCA
GUC.GCGAACUC....AAGCGCCCUCGA...CAGC.....CAC..ACG....GCUG....GUCGG..GGGCUUUUGUUUUA
NZ_AQUW01000005.1/89715-89581
AUGACCAAC...CUUCGACUUGUACUCGUA...AUU..ACCG.AGCGGCGCGCGCCG.........................UAA.CGGUA................CGUAC.UA.
GUC.GUCCCAUACAGCACGCGCCCUCGA..CGGACUCGAACAC..CAACCGAGUUC..CCGCCGA..GGGUUUUGGUUUAU
NZ_LDYE01000003.1/12036-12157
AUGAACAUU...AUUGGACUUGUACUU......GUA..ACCG.CGCGGCGUUUGCCG.........................UGA.CGGACU............ACUUUUAC.UA.
GUC.CUCAUCGC....AAACGCCU..CA...CCGCAC..ACAG..ACA..GUGCGG...CG..GA..GGGCGUUUCUUAUA
NC_021915.1/1289804-1289938
AUGAUCAUU...UCGCGACUUCAGGUACUA...GUA..GUAG.CUCGGCGCUUGCCG.........................UAG.CGGCUGA.........CCCACACCUG.CA.
GUC.GUCCCGGU....AAGCGCCCUCGA..AUAGCAC..CCACCCAGA..GUGCUA...CAUCGA..GGGUUUUUGCCGUU
SRS053917_LANL_scaffold_39801/111504-111377
AUGAACAUC...AUUCGACUUGUGCUAGUA...GUA..UCCGCCACGGCGCCUGCCGUUU.CGGCUUUC.............UAG.........................UC.AA.
GUC.GAUCACGU...CAAGCGCCCUCGG...UCACAC...CAC..A.A..GUGUGG...AUUCGG..GGGCUUUUGCAUUA
NZ_LKST01000003.1/367238-367360
AUGCUCAUU...AUUCGACUUCUCCUU......GUA..CCCG.AGCGGCGCGUGCCG.........................UAG.CGGCC................AGUUG.AG.
GUC.GAAGAGCA.....AGCGCCCUCCG...CAACAC..CCACCCACG..GUGGAU...GCGCGA..GGGCGCUUCGCAUA
NZ_AQXB01000005.1/82875-82752
AUGCUGAUU...AUUCGACUCGUACUU......GUA..CCCG.GGCGGCGCGUGCCG.........................UAG.CGGCU................GGUUC.AG.
GUC.GAAGCGCA....CGGCGCCCUCCG...CAACAC..CCACCCACG..GUGGCU...GCGCGA..GGGCGCUUCGCAUA
NZ_LKEV01000003.1/168206-168329
AUGCUUAUU...AUUCGACUUGUACUC......GUA..CCCG.AGCGGCGCGUGCCG.........................UAA.CGGCU................GGUUC.AA.
GUC.GAAGCGCA....CGGCGCCCUCCG...CAACAC..CCACCCACA..GUGGCU...GCGCGA..GGGCGUUUUGCAUA
NZ_CP011545.1/1146439-1146542
AUGAUCAAC...AUUCGACUCGUGGUA......AUU..ACC...UAA.CGGCGCUCGCCGCUU.CGGCCUUACC.CA.
GUC.GUCUCGAC....AAGCGCCCUCGA..CUAGCAC..CCACCAUUA..GUG.CU.........................
SRS045313_C1283849/196-316
AUGACCAUU...AUUCGACUUGUACUC......GUA..CGCC.CUCGGCGCUUGCCG.........................UAA.CGGU.................UAUAC.AA.
GUC.GUUUCGUC.....AGCGCCCUCGG...AAGCAC.CACAC.CACACGGUGC......CUCGG..GGGCUUUGAUUUUU

SRS015574_C3315556/319-199
AUGACCAUU...AUUCGACUUGUACUC......GUA..AGCC.CUAGGCGCUUGCCG.........................UAA.CGGU.................UAUAC.AA.
GUC.GUUUCGUC.....AGCGCCCUCGG...AAGCAC.CACAC.CACACGGUGC......CUCGG..GGGCUUUGAUUUUU
NZ_KB290822.1/138005-137885
AUGACCAUU...AUUCGACUUGUACUC......GUA..AGCC.CUCGGCGCUUGCCG.........................UAA.CGGU.................UAUAC.AA.
GUC.GUUUCGUC.....AGCGCCCUCGG...AAGCAC.CACAC.CACACGGUGC......CUCGG..GGGCUUUGAUUUUU
SRS018157_C4705301/300-180
AUGACCAUU...AUUCGACUUGUACUC......GUA..AGCC.CUCGGCGCUUGCCG.........................UAA.CGGU.................UAUAC.AA.
GUC.GUUUCGUC.....AGCGCCCUCGG...AAGCAC.CACAC.CACACGGUGC......UUCGG..GGGCUUUGAUUUUU
NZ_KB902182.1/38544-38414
AUGAUCAUU...UUACGACUAGUGGUA......GUA..CCGA.CGCGGCGCCUGCCG.........................UGA.CGGUGAG.........AACUUACUUC.UC.
GUC.GUUGUGAC....AAGCGCCCUCGA...CAGCAC..CCACCCAUA..GUGCCU...GUGCGA..GGGUUUUGUUAUGU
NZ_CP011312.1/880765-880888
AUGAACAUU...AUUCGACUUGUAGUG......AUU..ACCG.CGCGGCGCUUGCCGGCC.GCGGCU...............UAA......................CCUAU.AA.
GUC.GUCAAUGU...CAAGCGCCCUCGG...AAGCAU...CAC..A.A..GUGCUU....CGCGA..GGGUUUUUUCAUGG
NC_004369.1/1432212-1432338
AUGACCUCA...AUUCGACCUGUAGUAAUC...GUA..GCUG.CGCGGCGUUUGCCG.........................UAA.CGGUUU...............UCUAC.AA.
GUC.GUCUCGUC....AAGCGCCCUCGA...CAGUAC..CCAC.CACA..GUGCUG...UUUCGA..GGGCUUUGUUGUGG
NZ_CP007790.1/1096555-1096680
AUGAUCAAC...AUUCGACUCGUGGUA......GUA..GCUG.CGCGGCGCCUGCCG.........................UAA.CGACUC...............GCUAC.CCG
GUC.GUAUCGAC....AAGCGCCCUCGA...CAGCAC..CCACCAUCA..GUGCCU...GUUCGG..GGGCUUUGUCAUUA
NZ_CP011542.1/1481877-1482004
AUGAACAUU...AUUCGACUUGUACUCGUG...GUA..ACCGGCGCGGCGCCUGCCG.........................UAA.CGGCUU............CAUAGUUC.AA.
GUC.GAAUCGUC....AAGCGCCCUCCU...UCACAC...CAC..A.A..GUGUAG...AAGCGA..GGGUUUUUGUUUUA
SRS015803_C2663061/939-1067
AUGAACAUU...CUUCAGCUUGUACUCGUG...GUA..AUUGUCGCGGCGCCUGCCGUGU.CAACUGCC.............UAG........................UGC.AA.
GUU.GAUUUGUC....AAGCGCCCUCGG..UUCACGC...CAC..A.A..GUGUUG...AAGCGA..GGGUUUUUGCAUAG
SRS065099_LANL_scaffold_85044/921-1049
AUGAACAUU...AUUCAGCUUGUCCUCGUG...GUA..AUUGUCGCGGCGCCUGCCGUGU.CAACUGCC.............UAG........................UGC.AA.
GUU.GAUUUGUC....AAGCGCCCUCGG..UUCACGC...CAC..A.A..GUGUUG...AAGCGA..GGGUUUUUGCAUAG
SRS045049_WUGC_scaffold_11366/319-191
AUGAACAUU...CUUCAGCUUGUACUCGUG...GUA..AUUGUCGCGGCGCCUGCCGUGU.CAACUGCC.............UAG........................UGU.AA.
GUU.GAUUUGUC....AAGCGCCCUCGG..UUCACGC...CAC..A.A..GUGUUG...AAGCGA..GGGUUUUUGCAUAG
NZ_CP009220.1/1264918-1265039
AUGACCAUU...AUUCGACUUGUAGUA......GUA..ACCG.CACGGCGCCUGCCG.........................UAA.CGGC.................CUUAC.AA.
GUC.GUCUCGUC....AAGCGCCCUCGA...CAACAC..UCAC.CACA..GUGUUG...GAACGA..GGGCUUUCUUGUUG
NZ_CP015622.1/1292205-1292326
AUGACCAUU...AUUCGACUUGUACUA......GUA..ACCG.CGCGGCGCCUGCCG.........................UAA.CGGC.................CUUCC.AA.
GUC.GUCUCGUC....AAGCGCCCUCGA...CAACAC..UCAC.CAUA..GUGUUG...GACCGA..GGGCUUUCUUGUUG

AglaG_contig15215/131-8
AUGAACACC...AUUCGACUUGUACUA......GUA..CCCA.CGCGGCGCCUGCCG.........................UAA.CGGCC................CUACC.AA.
GUC.GUCCCGUC....AAGCGCCCUCGA...CAGCAC..CCACCACAU..GUGCUG...CUUCGA..GGGCUUUCUCGUUG
NZ_JNKV01000009.1/25614-25494
AUGAACAUU...AUUCGACUUGUGGUA......CUA..CCCG.CGCGGCGCCUGCCG.........................UAA.CGGCUC...............ACCUC.AA.
GUC.GUGUCGUC....AAGCGCCCUCGU...CAGCAC...CAC..A.A..GUGCCU...GAGCGG..GGGUUUUUGUCGUC
NC_017317.1/1027861-1027976
AUGAACAUU...AUUCGACUUGUAGUG......CUA..UCCG.AGCGGCACCUGCCG.........................UAA.CGG..................CCACC.AA.
GUC.GUAACGUA.....AGCGCCCUCGC...CAGCAU...CAC..A.A..GUGCUG....AUCGG..GGGCUUUUUUCGUA
NZ_LJVH01000019.1/275050-275166
AUGAACAUU...AUUCGACUUGUAGUG......CUA..UCCG.AGCGGCGCCUGCCG.........................UAA.CGG..................CCACC.AA.
GUC.GUAACGUA.....AGCGCCCUCGC...CAGCAU...CAC..A.A..GUGCUG...AUCGGG..GGGCUUUUUUCGUA
NZ_CP013261.1/973266-973382
GUGAACAUU...AUUCGACUUGUAGUG......CUA..CCCG.AGCGGCGCCUGCCG.........................UAG.CGG..................CCAUC.AA.
GUC.GUAACGUA.....AGCGCCCUCGC...CAGCAU...CAU..A.A..GUGCUG...AUCGGG..GGGCUUUUUUUCGU
NZ_CP011541.1/1113987-1114106
AUGAACAUU...AUUCGACUUGUACUC......GUA..ACCU.CACGGCGCUUGCCG.........................UAG.GCGAC................GCUAC.AA.
GUC.GACCCCGC....AAGCGCCCUCGA...CAGCAC..ACAC..A.A..GUGCUG....GCCGG..GGGCUUUUGCUUUA
NZ_MKAX01000239.1/151054-151175
AUGAUCAAC...CGGCGACUUGUAAUU......GUA..CCCA.CGCGGCGCUUGCCG.........................UAG.CGGUAC............CG.UUUCC.AC.
ACC.GCCGAUUC....AAGCGCCCUCGA...CAGCAC....AC..AAC..GUGCUC....GCCGG..GGGCUUUUGUUUUG
NZ_KV787969.1/105225-105104
GUGAUCAAU...CAGCGACUUGUAAUUAUU...GUA..CCCA.CGCGGCGCUUGCCG.........................UAG.CGGGUU............CGUUUUAC.AC.
GCC.GCAGACAC....AAGCGCCCUCGA...GAGC......AC..ACC....GCUC....GCCGG..GGGCUUUUAUUUUU
NZ_KV827986.1/43957-44078
GUGAUCAAU...CAGCGACUUGUAAUUAUU...GUA..CCCA.CGCGGCGCUUGCCG.........................UAG.CGGGUU............CGUUUUAC.AC.
GCC.GCAGACAC....AAGCGCCCUCGA...GAGC......AC..ACC....GCUC....GUCGG..GGGCUUUUAUUUUU
NZ_KV825973.1/51879-51759
GUGAUCAAU...CAGCGACUUGUAAUUAUU...GUC..CCCA.CGCGGCGCUUGCCG.........................UAG.CGGGUU............CGUUUUAC.AC.
GCC.GCAGACAC....AAGCGCCCUCGA...GAGC......AA..A.C....GCUC....ACCGG..GGGCUUUUAUUUUU
NZ_CP009215.1/1026773-1026898
GUGAUCAAC...GAGCGACUUGUAAUUAUC...GUA..UCCC.GACGGCGCUUGCCG.........................UAG.CGGGUG............CGUUUUAC.UAC
GCC.GCUCACAGA.ACAAGCGCCCUCGA...GAGC......UC..GAC....GCUC....GUCGG..GGGCUUUUCUUGUU
NZ_CBYN010000065.1/9457-9339
GUGAUCAAC...CAGCGACUCGUCAUUCUC...GUA..CCCU.CUCGGCGCUUGCCG.........................UAG.CGGGAU............CGUUUGAC.AC.
GCC.GCAUACGA.....AGCGC.CUCGA...GAGC......AC..A.C....GCUC....CGCGG..GCGCUUUUUUAGUU
NZ_CP009211.1/1108014-1108142
AUGAUCAAC...CAGCGACUAGUAGUACGA...AUU..CCCA.CGCGGCGCUUGCCG.........................UAG.CGGGUU............CGUUUUCC.UAC
GCC.GCUGACAA...CAAGCGCCCUCGA...CAGCAC..CUAC..C....GUGCUG...AUUCGG..GGGCUUUUGUUUUG

```
NZ_KV818028.1/1883-2013
AUGAUCAAC...CAGCGACUAGUAGUACGA...AUU..CCCA.CGCGGCGCUUGCCG.........................UAG.CGGGUU............CGUUUUUC.UAC
GCC.GCUGAAACA.ACAAGCGCCCUCGA...CAGCAC..ACAC..C....GUGCUG...AUUCGG..GGGCUUUUGUUUUG
#=GC SS_cons                                      :::::::::...,,<<<<<<<<--------...---..<<<<.-
<<<<<____>>>>.........................>--.>>>>--............------->.>>.>>>.>>------....,,,-
<<<<<<<<...<<<<<<..____..___..>>>>>>...-->>>>..>>>>,,,:::::::
#=GC R2R_LABEL
sss...............UUU...UUU......UUU...............UUU............................ttt...............................
..................aaaaaaa........................................................
#=GC R2R_XLABEL_antiterminator
............................................aaaaaaa.................................................................
..................aaaaaaa........................................................
#=GC R2R_XLABEL_ticks
.S.................U.....U........U..............A..U..............................T................................
...................A.............................................................
#=GC R2R_XLABEL_test_antiterminator
............................................aaaaaaa.................................................................
..................bbbbb..........................................................
#=GC R2R_XLABEL_flanks
...b.o..............................................................................................................
............................................................................e....
#=GC R2R_XLABEL_var
...........................3....4.........................................................1...................2.....
...................................a...............b.............................
#=GF R2R outline_along_backbone t rgb:0,0,0
#=GF R2R outline_along_backbone s rgb:0,0,0
#=GF R2R outline_nuc U
#=GF R2R outline_nuc antiterminator:a
#=GF R2R tick_label ticks:T Stop
#=GF R2R tick_label ticks:S start
#=GF R2R tick_label ticks:U I/L/V
#=GF R2R place_explicit flanks:b flanks:b-- 45 1 0 0 0 90
#=GF R2R place_explicit flanks:o flanks:o-- -45 1 0 0 0 -90
#=GF R2R place_explicit flanks:e flanks:e-- -45 1 0 0 0 -90
#=GF R2R var_backbone_range var:1 var:2
#=GF R2R var_backbone_range var:3 var:4
#=GF R2R SetDrawingParam varHairpinNumFakePairs 2
#=GF R2R var_hairpin var:a var:b
```

#=GF DUPLICATES NZ_JNKV01000009.1/25614-25494=NZ_CP004353.1/1319708-1319828 NZ_JVVY01000009.1/43665-
43534=NZ_JVSN01000055.1/30747-30616=NZ_JVSI01000060.1/137982-138113=NZ_AQXC01000002.1/192215-
192346=SRS017044_C178023/688-819 NZ_CP017639.1/1290196-1290067=NZ_KV789254.1/82790-82661=NZ_LT622815.1/377667-377796
SRS015044_WUGC_scaffold_38868/315-195=SRS016360_C2585069/108-228=SRS047634_LANL_scaffold_134194/810-930
NZ_JUOH01000021.1/24827-24716=NZ_JUZU01000012.1/26996-27107 NZ_ATVF01000002.1/139928-139807=NC_020506.1/1252789-
1252910 NZ_GL397138.1/1172545-1172653=NZ_GG666998.1/364403-364511=NZ_KI515712.1/1223242-
1223350=NZ_KI515704.1/381703-381811=NZ_KI515710.1/117675-117567=NZ_KI515760.1/404867-404975=NZ_KI515719.1/381432-
381540=SRS013876_WUGC_scaffold_1004/330-222=SRS015937_WUGC_scaffold_2433/9700-
9808=SRS015996_WUGC_scaffold_10113/131-239=SRS047225_C228367/212-104=SRS056906_LANL_scaffold_14970/18578-18686
NZ_JRUB01000003.1/93397-93266=NZ_CP011311.1/1097943-1098074 NZ_KI515742.1/108057-107949=NZ_KI515730.1/374415-
374523=NZ_KI515775.1/375122-375230=SRS014901_C259564/386-278=SRS017820_C154801/497-
605=SRS022006_Baylor_scaffold_1889/151-43=SRS044474_C227715/151-43 NZ_MLCQ01000018.1/86330-
86458=NZ_MLCR01000009.1/25681-25809 NZ_JVTN01000036.1/102026-102165=NZ_JVTL01000070.1/82688-
82827=NZ_JVBA01000133.1/5876-5737=NZ_JULW01000106.1/5851-5712=NZ_JUSN01000054.1/74334-74473=NZ_JULV01000128.1/5848-
5709=NZ_JVCW01000039.1/145752-145891=NZ_KV788492.1/102047-102186=NZ_KV788319.1/5984-5845=NZ_LAYR01000003.1/255152-
255291 NZ_ACVP01000012.1/205927-206038=NZ_GL542875.1/94639-94528 NZ_KV812479.1/16371-16504=NZ_KV816288.1/24900-24767
NZ_CAFW01000088.1/35810-35682=NZ_CP004350.1/1819709-1819581 NZ_KV805153.1/141775-141907=NZ_LAYQ01000012.1/237432-
237564 NZ_JIAH01000006.1/288993-289124=NZ_KI515715.1/2015671-2015540=NZ_KI515718.1/292645-
292776=SRS013876_WUGC_scaffold_1403/18882-18751 NZ_AFYA01000002.1/29005-28884=NZ_AKXP01000019.1/4612-
4733=NZ_AGQQ02000002.1/1601582-1601703=NZ_AQPS01000033.1/4611-4732=NZ_LOQW01000020.1/139507-
139386=NZ_LOQS01000038.1/182360-182481=NZ_LOQX01000021.1/135585-135706=NZ_LOQT01000027.1/139573-
139452=NZ_LOQV01000011.1/14024-14145=NZ_LOQY01000042.1/190312-190433=NC_020519.1/1337839-
1337960=NC_021352.1/1531394-1531515=NC_021351.1/1531395-1531516=NC_022040.1/1340736-1340857=NZ_CP010451.1/1407901-
1408022=NZ_JXBH01000015.1/135617-135738=NZ_CP007722.1/1422941-1423062=NZ_CP007724.1/1392124-
1392245=NC_006958.1/1339309-1339430=NZ_CP011309.1/1416143-1416264=NZ_CP012194.1/1481996-
1482117=NZ_CP013991.1/1440461-1440582=NZ_CP014984.1/1408353-1408474=NZ_CP004062.1/1457045-
1457166=NZ_CP004046.1/1458294-1458415=NZ_CP016335.1/1439742-1439863=NC_003450.3/1337840-
1337961=NZ_JYEG01000003.1/135627-135748=NC_009342.1/1480626-1480747=NZ_CP018175.1/1450060-1450181
NZ_KV794854.1/187802-187670=NZ_KV817269.1/138421-138553 NZ_BCRC01000002.1/59853-59980=NZ_JSEF01000012.1/59924-60051
NZ_JUMN01000085.1/2198-2330=NZ_KV805024.1/7626-7758=NZ_KV794798.1/110983-110851=NZ_KV818338.1/109252-
109120=NZ_KV821964.1/4616-4748=NZ_KV827349.1/19880-20012=NZ_KV796767.1/26731-26599 NZ_ACSH02000008.1/90454-
90326=NZ_EQ973332.1/89274-89146=SRS011098_C1888314/456-328=SRS011152_Baylor_scaffold_32214/76-
204=SRS011343_Baylor_scaffold_9124/68942-68814=SRS012285_Baylor_scaffold_38889/3444-3316=SRS013170_C4232195/334-
462=SRS013723_Baylor_scaffold_19554/13513-13641=SRS013949_C3544868/373-501=SRS013950_C2148140/467-
339=SRS014473_C1091495/257-129=SRS014476_C3457652/1285-1413=SRS014477_C1645836/1779-
1907=SRS014578_WUGC_scaffold_51652/938-1066=SRS014690_WUGC_scaffold_24606/456-584=SRS014691_C2690067/439-
567=SRS014894_WUGC_scaffold_19927/38168-38296=SRS015060_C1523053/249-377=SRS015063_WUGC_scaffold_29229/836-
708=SRS015158_WUGC_scaffold_22078/836-708=SRS015215_WUGC_scaffold_4516/833-705=SRS015278_C1850386/706-
834=SRS015378_WUGC_scaffold_5660/6432-6304=SRS015440_WUGC_scaffold_14348/2115-2243=SRS015470_C3258071/169-

297=SRS015574_WUGC_scaffold_3172/62405-62277=SRS015755_WUGC_scaffold_19812/865-993=SRS015989_C2173651/1946-
2074=SRS016043_WUGC_scaffold_40608/56137-56265=SRS016200_WUGC_scaffold_42202/1460-
1588=SRS016331_WUGC_scaffold_13495/137-265=SRS016360_Baylor_scaffold_6909/3814-
3686=SRS016575_Baylor_scaffold_47591/3624-3496=SRS016746_Baylor_scaffold_88654/1950-
2078=SRS017025_Baylor_scaffold_28510/231-103=SRS017139_C3698162/331-459=SRS017304_Baylor_scaffold_30957/3453-
3325=SRS017445_C3182885/279-151=SRS017511_C3568662/1128-1256=SRS018337_C2391352/941-
813=SRS018665_WUGC_scaffold_20970/4137-4009=SRS019028_WUGC_scaffold_61507/452-324=SRS019128_WUGC_scaffold_17107/373-
501=SRS019225_WUGC_scaffold_32628/1157-1029=SRS019387_WUGC_scaffold_44091/1857-1985=SRS019591_C1936094/258-
130=SRS019906_WUGC_scaffold_19047/465-593=SRS019980_Baylor_scaffold_25718/3565-
3437=SRS020226_Baylor_scaffold_45329/1744-1872=SRS020862_C941459/1792-1920=SRS021477_Baylor_scaffold_55202/6515-
6387=SRS021960_C2207788/270-142=SRS022083_Baylor_scaffold_2182/63720-63592=SRS022149_C2841960/37-
165=SRS023595_Baylor_scaffold_97314/1121-993=SRS023938_Baylor_scaffold_12344/39709-
39581=SRS023964_Baylor_scaffold_3119/63325-63197=SRS024021_Baylor_scaffold_31203/17120-16992=SRS024144_C1813426/641-
513=SRS024289_LANL_scaffold_20158/62721-62593=SRS024447_C3962488/126-254=SRS024561_LANL_scaffold_7110/1946-
2074=SRS024649_LANL_scaffold_34541/703-575=SRS042984_LANL_scaffold_75089/1640-
1768=SRS043018_WUGC_scaffold_48238/5141-5013=SRS043772_WUGC_scaffold_18378/813-
941=SRS047100_WUGC_scaffold_16374/3741-3613=SRS049268_LANL_scaffold_9520/22534-
22662=SRS051244_LANL_scaffold_48275/238-110=SRS051378_LANL_scaffold_29437/771-643=SRS051930_LANL_scaffold_1281/1308-
1180=SRS052604_LANL_scaffold_9342/9475-9347=SRS052876_LANL_scaffold_51045/641-513=SRS053584_LANL_scaffold_28301/126-
254=SRS054653_LANL_scaffold_31340/16625-16497=SRS055378_LANL_scaffold_96117/1950-
2078=SRS055450_LANL_scaffold_52702/132-4=SRS058053_LANL_scaffold_74769/238-110=SRS058808_LANL_scaffold_23656/1798-
1926=SRS063215_C2207611/134-262=SRS063603_LANL_scaffold_40849/1011-883=SRS063932_LANL_scaffold_50586/1876-
1748=SRS065310_C1358931/1128-1256=Buccal_mucosa_LANL_scaffold_140899/331-459=SRS015064_C1733276/1285-
1413=SRS022602_C4050641/1286-1158=SRS044366_C950260/1823-1951=SRS053630_LANL_scaffold_7039/2003-2131
NZ_KV788396.1/85810-85934=NZ_KV835927.1/135683-135807 NC_004369.1/1432212-1432338=NZ_GG700686.1/133170-133296
SRS018157_Baylor_scaffold_43439/431-303=SRS018394_Baylor_scaffold_32155/126-254 NZ_BCSU01000002.1/86538-
86657=NZ_CP005286.1/1155059-1155178 NZ_KB290822.1/138005-137885=SRS011098_Baylor_scaffold_17209/2160-
2040=SRS011126_Baylor_scaffold_56581/387-507=SRS011152_C4255914/159-279=SRS011255_Baylor_scaffold_25511/2580-
2460=SRS012285_Baylor_scaffold_56424/3240-3360=SRS013170_Baylor_scaffold_40316/36547-
36427=SRS013533_PGA_scaffold_40454/1176-1056=SRS013949_WUGC_scaffold_11817/951-1071=SRS014476_C3417561/65-
185=SRS014894_WUGC_scaffold_57940/4417-4297=SRS015063_WUGC_scaffold_40034/918-798=SRS015158_C2599853/339-
459=SRS015278_C1854776/109-229=SRS015378_C1042608/728-608=SRS015440_WUGC_scaffold_60932/3542-
3662=SRS015470_WUGC_scaffold_40685/3240-3360=SRS015650_WUGC_scaffold_13879/1144-
1264=SRS015755_WUGC_scaffold_44004/5499-5619=SRS015803_WUGC_scaffold_49229/1213-
1093=SRS015899_WUGC_scaffold_43167/6218-6338=SRS015989_WUGC_scaffold_11868/8650-8770=SRS016043_C2161539/155-
275=SRS016092_WUGC_scaffold_31986/1185-1305=SRS016200_C2214117/946-1066=SRS016331_C4180843/151-
271=SRS016746_Baylor_scaffold_8565/27510-27630=SRS017025_Baylor_scaffold_34925/613-
493=SRS017139_Baylor_scaffold_94023/2038-1918=SRS017227_C5168493/159-279=SRS017304_C1940726/121-
1=SRS017445_C3165753/289-169=SRS017511_Baylor_scaffold_30269/29854-29974=SRS017691_C4988944/145-

265=SRS018394_Baylor_scaffold_68124/3475-3595=SRS018573_WUGC_scaffold_40370/1015-895=SRS018665_C3758973/465-
345=SRS018778_C1280603/2955-3075=SRS018975_WUGC_scaffold_39585/779-899=SRS019077_C2668993/171-
51=SRS019128_C4401874/155-275=SRS019225_WUGC_scaffold_23966/925-805=SRS019333_WUGC_scaffold_24063/886-
766=SRS019387_C1729534/159-279=SRS019591_WUGC_scaffold_40888/740-860=SRS019980_Baylor_scaffold_24955/837-
717=SRS020340_C2981410/820-940=SRS020862_C926752/490-370=SRS021477_Baylor_scaffold_44844/1305-
1185=SRS021960_C2220814/727-607=SRS022536_LANL_scaffold_63292/877-997=SRS022725_LANL_scaffold_46254/1270-
1390=SRS023538_Baylor_scaffold_21013/670-790=SRS023595_C7197952/802-922=SRS023964_Baylor_scaffold_57922/17838-
17718=SRS024021_Baylor_scaffold_11826/3550-3430=SRS024289_LANL_scaffold_59783/606-
486=SRS024561_LANL_scaffold_23875/257-377=SRS024649_C3692425/893-1013=SRS042984_LANL_scaffold_34447/4204-
4084=SRS043018_WUGC_scaffold_13057/1501-1381=SRS043755_WUGC_scaffold_44073/595-715=SRS043772_C1800500/155-
275=SRS045197_C3572388/221-101=SRS047100_C2034960/305-185=SRS047265_WUGC_scaffold_11415/306-
186=SRS049268_LANL_scaffold_66564/3240-3360=SRS051930_C3033858/150-270=SRS051941_C4083639/1253-
1373=SRS052876_C2251838/137-17=SRS053584_LANL_scaffold_40382/1548-1428=SRS054430_C2053627/94-
214=SRS054653_LANL_scaffold_32230/918-798=SRS055378_C4043811/361-481=SRS055401_C2324768/595-
715=SRS058053_C5447317/595-715=SRS058808_C2874116/232-112=SRS063215_C2265232/550-
670=SRS063999_LANL_scaffold_68566/733-613=SRS064449_C4128646/382-262=SRS064493_LANL_scaffold_27106/2697-
2817=SRS065099_LANL_scaffold_21269/1650-1770=Buccal_mucosa_LANL_C65166083/316-196=SRS015064_C1735859/1255-
1375=SRS022602_Baylor_scaffold_113244/498-618=SRS053630_LANL_scaffold_21470/4775-4895=SRS053917_C3828842/670-
790=Subgingival_plaque_LANL_scaffold_26684/179-299=Supragingival_plaque_LANL_scaffold_376229/382-262
NZ_LTEB01000025.1/84190-84331=NZ_KV789226.1/24967-24826=NZ_KV788623.1/124068-124209=NZ_KV793700.1/24457-
24316=NZ_KV800712.1/203374-203233=NZ_KV827729.1/200876-200735 NZ_JAQQ01000012.1/73281-73397=NZ_AJGI01000001.1/12616-
12500=NZ_AJVH01000012.1/316248-316364=NZ_AUZN01000065.1/16043-16159=NZ_MLBM01000001.1/78685-
78569=NZ_MLBL01000001.1/47202-47086=NZ_MLBK01000001.1/47296-47180=NZ_MLBH01000001.1/78684-
78568=NZ_MLBN01000001.1/47301-47185=NZ_MLBI01000007.1/16600-16716=NZ_MLBJ01000004.1/92334-
92450=NZ_MIOA01000012.1/53908-54024=NZ_MINX01000052.1/34703-34587=NZ_MIOI01000001.1/34703-
34587=NZ_MIOK01000004.1/34702-34586=NZ_MION01000050.1/54077-54193=NZ_MIOO01000007.1/53976-
54092=NZ_MINZ01000025.1/53885-54001=NZ_MIOJ01000006.1/34703-34587=NZ_MIOC01000012.1/86767-
86651=NZ_MIOD01000034.1/34703-34587=NZ_MIOL01000016.1/34702-34586=NZ_MIYQ01000065.1/38046-
38162=NZ_MIOF01000045.1/314610-314726=NZ_MIYR01000032.1/87293-87177=NZ_MINY01000011.1/53891-
54007=NZ_MIOB01000007.1/46470-46586=NZ_MIOE01000034.1/53880-53996=NZ_MIYO01000033.1/53881-
53997=NZ_MIYP01000077.1/11629-11513=NZ_MIOM01000045.1/34702-34586=NZ_MIOP01000023.1/330986-
331102=NZ_MIOR01000094.1/34703-34587=NZ_MIOQ01000083.1/53893-54009=NZ_MIYN01000029.1/53891-
54007=NZ_MIOH01000019.1/54049-54165=NZ_MIOG01000034.1/53881-53997=NZ_MIYS01000054.1/83145-
83029=NZ_MKYM01000001.1/88424-88308=NZ_MKYG01000002.1/87244-87128=NZ_MKYI01000001.1/46983-
46867=NZ_MKYN01000016.1/53889-54005=NZ_MKYL01000006.1/53884-54000=NZ_MKYH01000003.1/87244-
87128=NZ_MKYJ01000002.1/104064-103948=NZ_MKYK01000001.1/64343-64227=NZ_AUZO01000013.1/73281-
73397=NZ_LJXS01000034.1/73466-73582=NZ_LJXR01000012.1/190773-190889=NC_016785.1/1031779-1031895=NC_016782.1/1029479-
1029595=NC_016787.1/1044455-1044571=NC_016799.1/1108986-1109102=NC_016783.1/1025857-1025973=NC_016786.1/1029452-
1029568=NC_016789.1/1095415-1095531=NC_016788.1/1054467-1054583=NC_016790.1/1021856-1021972=NC_016801.1/1042317-

1042433=NC_002935.2/1081747-1081863=NC_016800.1/1083864-1083980=NC_016802.1/1051105-1051221=NZ_LN831026.1/1006434-
1006550=NZ_CP018331.1/272814-272930=NZ_JRUZ01000001.1/92218-92102=NZ_JZUJ01000002.1/259364-
259480=NZ_JAQN01000016.1/314671-314787=NZ_JAQP01000016.1/73281-73397=NZ_JAQO01000008.1/190817-190933
NZ_AQUX01000009.1/35144-35016=NZ_CP006764.1/1252734-1252862 NZ_CP010827.1/1294698-1294827=NZ_KV788209.1/202054-
201925 NZ_LXGG01000012.1/107049-106925=NZ_HG001322.1/342464-342588 NZ_JVXO01000045.1/2686-2576=NZ_KI515722.1/103383-
103273=NZ_KI515735.1/1452474-1452584=NZ_KI515731.1/2096923-2096813=NZ_KI515752.1/359342-359452=NZ_KI515767.1/355261-
355371=SRS058213_LANL_scaffold_6881/468-358=Anterior_nares_Baylor_scaffold_15594/114-
4=R_ear_crease_WUGI_scaffold_82427/5-115 NZ_KV806831.1/110751-110618=NZ_KV804013.1/186952-
186819=NZ_KV799871.1/18382-18515=NZ_KV797592.1/188408-188275=NZ_KV802435.1/16344-16477=NZ_KV805178.1/34466-
34333=NZ_KV817117.1/111876-111743 NZ_CP013699.1/1904309-1904194=NZ_CP014341.1/1903945-1903830
NZ_LDYD01000008.1/204736-204866=NZ_KB892439.1/33352-33222 AglaG_contig15215/131-8=AglaG_GBVDVLI01EC2ZM/131-
8=AglaG_GDN60OX02JD4NE/253-376 NZ_KQ959770.1/30883-31013=NZ_KQ961710.1/14886-14756=NZ_LT596208.1/742899-
743029=NZ_KV796382.1/30876-31006=NZ_KV801723.1/14886-14756=NZ_KV817199.1/14882-14752=NZ_KV818618.1/30881-31011
NZ_JPJB01000001.1/90561-90446=NZ_MDWN01000003.1/328327-328442=NC_014329.1/946715-946830=NC_017303.1/946526-
946641=NC_017307.1/940031-940146=NC_017305.1/946526-946641=NC_017308.1/924291-924406=NC_017306.1/946706-
946821=NC_016781.1/946706-946821=NC_017031.1/946549-946664=NC_016932.1/934812-934927=NZ_CP012136.1/973372-
973487=NC_017462.1/946513-946628=NC_018019.1/929696-929811=NZ_CP008924.1/1484025-1484140=NZ_CP008922.1/1500622-
1500737=NZ_CP008923.1/95711-95596=NZ_CP009927.1/946653-946768=NC_017301.1/946331-946446=NC_017300.1/946609-
946724=NZ_CP010889.1/946622-946737=NZ_CP011474.1/946665-946780=NZ_CP010795.1/1906775-1906660=NZ_CP012022.1/952953-
953068=NZ_CP012695.1/1905333-1905218=NZ_CP012837.1/1903290-1903175=NZ_CP013146.1/1906078-
1905963=NZ_CP013327.1/946818-946933=NZ_CP013697.1/1905687-1905572=NZ_CP013698.1/1905669-
1905554=NZ_CP013263.1/1247488-1247373=NZ_CP013262.1/325931-326046=NZ_CP013260.1/1078693-
1078808=NZ_CP014543.1/1905778-1905663=NZ_CP015309.1/937871-937986=NC_017730.3/1008967-1009082=NZ_CP015100.1/946664-
946779=NZ_CP016826.1/1906058-1905943=NZ_CP015192.1/1009009-1009124=NZ_CP015187.1/1008942-
1009057=NZ_CP015185.1/1008957-1009072=NZ_CP015184.1/1008940-1009055=NZ_CP015190.1/972262-
972377=NZ_CP015191.1/1008931-1009046=NZ_CP015189.1/972167-972282=NZ_CP015183.1/1008939-
1009054=NZ_CP015186.1/1008953-1009068=NZ_CP015188.1/1008941-1009056=NZ_CP017384.1/822498-
822613=NZ_CP017292.1/973231-973346=NZ_CP017291.1/973145-973260 NZ_LZMN01000041.1/68611-68480=SRS053437_C430641/624-
755 NZ_KV787969.1/105225-105104=NZ_KV787065.1/76623-76744=NZ_KV789315.1/55716-55595=NZ_KV801051.1/95117-
94996=NZ_KV803842.1/143390-143511=NZ_KV821352.1/98958-98837=NZ_KV826421.1/78205-78326=NZ_KV833343.1/144826-144947
NZ_JIAJ01000008.1/123276-123400=NC_020302.1/1402578-1402702 NZ_ACLH01000005.1/107419-107286=NC_012590.1/1251677-
1251810=NZ_KV788191.1/41387-41254=NZ_KV803645.1/18536-18669=NZ_KV814384.1/18592-18725=NZ_KV818446.1/193153-193020
NZ_LGSX01000012.1/4538-4653=NZ_LGSY02000112.1/20322-20437=NZ_MIOS01000003.1/72653-72768=NZ_AYUJ01000003.1/335453-
335568=NC_015683.1/1030704-1030819=NZ_CP009583.1/1017240-1017355=NZ_CP009500.1/1014058-
1014173=NZ_CP009622.1/1011358-1011473=NZ_CP009716.1/1022115-1022230=NZ_CP011913.1/1058461-
1058576=NZ_CP010818.1/1013803-1013918=NC_018101.1/1101954-1102069 NZ_CP012342.1/904626-904498=NZ_KV786473.1/24533-
24405
//

File S2. Sequence alignment of the *ilvB*-OMG motif.

```
# STOCKHOLM 1.0
#=GF END_OF_NEW_HITS here
#=GF DUPLICATES NONE
#=GF SORT_STATS 43 274 0
NZ_JXQW01000032.1/44045-44253                    UUGCAAUCGGAACGGCCAA-GAUGUCGACGAUCAAUUU-UGGCCC-CGACCUUCGUGCCGU-CG-CG-
AAGAUCGAGGCGAUUCCGA....UGA.................................Ucc.u.gc.c-G..CAUGGUCAAGCACGUGACC------GGGCUA..-
CGA..............UUCGCUGCCGUCG-CGCGGGUC-A---CCGACAAACAGUGG---AUCACCUGCGCGGUCG------ACGACU---CGAUCGAGUU---CGGGC--
UCAAGCCCG--GCGA
NZ_CM002299.1/1278545-1278763                    AUG...........GC-AACGCGGAGUACCCUCCGCCUCUGGCCC-CUGUUUCGGUGCGAU-UAUCC-
GAGA---................UAG.....................CUGUUCUUGCGUCCC.G.UC--GG..-CGGUAC--CUU-GCGACCUAUU--GCGCAAA--GUA----
CGGCAGUCCACACUGCGUUACCG-CGCAGGGCGA---CAGCG--ACACGGGGUUCCUUCGUG----CUUGU---UCUCCCUC---UG---UUUUU---UGCGG--UUGACCGGA-
UGUGU
NZ_DS999408.1/460137-460354                      AUG.....GUAACGGC-AAUGCAGAGUACCCUCUGCCCCUGGCCC-CUAUCCCGGUGCGAU-UAUCC-
GGUU---................UAG.....................CUGUACUUGCGUCCC.G.UC--GG..CCGUGGU---UU-GUCACUCAUU--GGACAAA--UCC----
CGGCAGUCCACACUGCGUUACCG-CGCAGGGCGA---CAACG--ACACGGGGUCCCUUCGUG----CUUGU---UAUCCCUC---UGUUCUUUUU---GCGGC------AUCUU-
UUUGU
NZ_KN234793.1/6429-6648                          GUG.....GCAACGGCUAGCGUCGGGUACCCCCGUCCUCUGGCCUACUGCAUCAACGGUUUCAG-UU-
GGUUCAGCUGCCCGCAUGUCCCGUAA..........................---.............---..---AACGGUGCU-GCCGCAUG-----GGUGGU--CGC--
CGUGGGUC-CGACACUGCGCUACCC-CGCAGGGCGA---C--AC--GCACGGG---UUCUCGUG----AUUGUCAUUCCCC---CUUUGGCGAGUAG---ACGCU-
GUUGUUGCGU-CCGGC
NZ_DS999411.1/263744-263953                      GUG.....................AGUACCCUCAUCCACUGGCCUACCCCCAUAACGGGA--...---
.......................UAG.....................UUAUGAGGGCUGUUUGC.G.GGUUCCGUCAGGU-GCGCCGCUCA.----...GGCGUU--ACU----
CGGGCU-CGACACUGCGUUACCU-CGCAGGGCGA---CCGAU--ACACGGG----UUUCGUG----AUCG------AUGUUC---CGGUGUGGUUACCCCGGC--GCGAUCCGG-
CCAGU
JGI20160J14292_10002764/11909-12124              AUG.....................AGUACCCUCAUCCUCUGGCCUACCUUAAUGACGGGA--...---
.......................UAG.....................UUAUUUGGGCUGUUUGC.G.GGUUCCGUCAGUGC.CUC.GUUUUUAGA--..AGCGAU-GCAC-
GUCCGGGCU-CGACACUGCGCUACCU-CGCAGGACGACCGCCGA---ACACGGG----UUUCGUG----AUCG------CUGUUC---UGUGAGUUAU---CCGGA--
GCGAUCCGG-CCAGU
2236876002_087674/165-382
AUG.....................AGUACCCUCAUCCUCUGGCCUACCUUAAUGACGGGA...............................UAG.....................U
UAUUUGGGCUGUUUGC.G.GGUUCCGUCAGUGC.CUC.GUUUUUAGA---.AGCGAU-GCAACGUCCGGGCU-CGACACUGCGCUACCUACGCAGGACGACCGCCGA---
ACACGGG----UUUCGUG----AUCG------CUGUUC---UGUGAGUUAU---CCGGA--GCGAUCCGG-CCAGU
NZ_AGIF02000001.1/396050-396262
GUG.....................AGUACCCUCAUCCUC....................................................UGACCCACCCUAAUGACGGGAUAGU
UAUUUGGGCUGUUUGC.G.GGUUCCGUCAGUGU.UGC.GCAGGUAUA---.UGCGUC-UCAC-GCUCGGGCU-CGACACUGCGCUACCU-CGCAGGACGG---CCGA---
ACACGGG----UUUCGUG----AUCG------AUGUUC---UGGAUGGUUU---CCGGA--UCGAUCCGG-CCAGU
```

84527787/189-401
AUG.....................AGUACCCUCAUCCUC...----
.UGACCCACCCUAAUGACGGGAUAGUUAUUUGGGCUGUUUGC.G.GGUUCCGUCAGUGU.UGC.GCAGGUAUA---.UGCGUC-UCAC-GCUCGGGCU-CGACACUGCGCUACCU-
CGCAGGACGG---CCGA---ACACGGG----UUUCGUG----AUCG------AUGUUC---UGGAUGGUUU---CCGGA--UCGAUCCGG-CCAGU
JCVI_READ_1101733453204/759-949   GUG.....................AGUACCCUCAUCCUC-----..-----
..........................--..-----.....UGACCCACCCUAAUGACGGGAUAGUUAUUUGGGCUGUUUGC.G.GGUUCCGUCAGUGU.UGC.GCAGUAUA---
..UGCGUC-UCAC-GCUCGGGCU-CGACACUGCGCUACU--CGCAGACGG-----CGA---ACACGGG----UUUCGUG---GAUCG------A-UGUC---UGGAUGGUUC---
C---------------------
JCVI_SCAF_1096627068406/1012-1224
GUG.....................AGUACCCUCAUCCUCUGGCCUACCUCUAUAACGGGA...............................UAG.....................U
UAUCUAGGCUGUUUGC.G.GGUUCCGUCAGUGUC.UCGCAGGGUUA---..UGUGAU-UCAC-GCUCGGGCU-CGACACUGCGCUACCU-CGCAGGGCGG---CCGA---
ACACGGG----UUUCGUG----AUCG------AUGUUC---UGGUAGGUUU---CCGGA--UCGGUCCGG-CCAGU
JCVI_READ_1101740177951/518-730
GUG.....................AGUACCCUCAUCCUCUGGCCUACCUUUAUAACGGGA...............................UAG.....................U
UAUUUAGGCUGUUUGC.G.GGUUCCGUCAGUGUC.UCGCAGGGUUA---..UGCGAU-UCAC-GCUCGGGCU-CGACACUGCGCUACCU-CGCAGGGCGG---CCGA---
ACACGGG----UUUCGUG----AUCG------AUGUUC---UGGUAGGUUU---CCGGA--UCGAUCCGG-CCAGU
JCVI_SCAF_1101668067822/655-867
GUG.....................AGUACCCUCAUCCUCUGGCCCACCUUUAUAACGGGA...............................UAG.....................U
UAUCUGGGCUGUUUGC.G.GGUUCCGUCAGUGUC.UAGCAGGUAUA.....UGUGACG.CAC-GCUCGGGCU-CGACACUGCGCUACCU-CGCAGGGCGG---CCGA---
ACACGGG----UUUCGUG----AUCG------AUGUUC---UGGUAGGUUU---CCGGA--UCGAUCCGG-CCAGU
NZ_AAVV01000015.1/20331-20535
AUG.....................AGUACCCUCAUCCGCUGGCCCACCCCCAUGACGGGA...............................UAG.....................U
UAUGAGGGCUGUUUGC.G.GGUUCCGUCUG.GUGC...GUUU--..---..GGC...-GCAC-U-UCGGGCU-CGACACUGCGCUACCC-CGCAGGCCGA---CCGAU--
ACACGGG----UUUCGUG----AUCG------CUGUUC---UGGCAGGGUG--CUAGGA-AUUGAUCCGA-CCAGU
DelMOSum2010_c10001046/9357-9561
AUG.....................AGUACCCUCAUCCGCUGGCCCACCCCCAUGACGGGA...............................UAG.....................U
UAUGAGGGCUGUUUGC.G.GGUUCCGUCUGGU......GCGUUAUG-----CGC.....AC----UCGGGCU-CGACACUGCGCUACCC-CGCAGGCCGA---CCGAU--
ACACGGG----UUUCGUG----AUCG------CUGUUC---UGGCAGGGUG--CUAGGA-UUUGAUCCGA-CCAGU
JGI20156J14371_10447667/68-272
AUG.....................AGUACCCUCAUCCUCUUGCCCACCCCCACAACGGGA...............................UAG.....................U
UGUGUGGGCUGUUUGC.G.GGUUCCGUAAUGU......GCGCUCAG-----UGC-----AC---CUCGGGCU-CGACACUGCGCUACCC-CGCAGGACGA---CCGAU--
ACACGGC----UUUCGUG----AUCG------AUGUUC---CGGUAUGGUAG-UCCGGA--AUGAUCCGG-CCAGU
JGI20156J14371_10041353/352-556
GUG.....................GGUACCCUCACCCUCUGGCCUACCUCCACAACGGGA...............................UAG....................-
UUGUGCGGGCUGUUUGC.G.GGUUCCGUAAUGU.....-GCGCUGUG-----CGC.....AC---CUCGGGCU-CGACACUGCGCUACCU-CGCAGGACGA---CCGAA--
ACACGGC----UUUCGUG----AUCG------GCGUUC---GGAUACGGUAG-UCCGGA--GCGAUCCGG-CCAGU
JGI20159J14440_10000898/6317-6521
GUG.....................GGUACCCUCACCCUCUGGCCUACCUUCACAACGGGA...............................UAG.....................U

```
UGUGCGGGCUGUUUGC.G.GGUUCCGUAAUGU....-GCGCUGUG-----CGC------AC.--CUCGGGCU-CGACACUGCGCUACCG-UGCAGGACGA---CCGAA--
ACACGGC----UUUCGUG----AUCG------GCGUUC---GGAUACGGUAG-UCCGGA--GCGAUCCGG-CCAGU
JCVI_SCAF_1096627426712/541-727
UUG..................GUAAGUACCCUUACCCUCUGGCCUACCUUCAGGACGGGA...............................UAG.....................U
UCUCAAGGCUGUUUGC.G.GGUUCCGUCAGGUAG...-AAGUUUAG-----CUCCU---ACU-GCUCGGGCU-CGACACUGCGCUACCG-CGCAGGUC-A---CCGAC--
ACACGGG----UUUCGUG----GUCG-------UGAUC---UGACGGGCG-------------------------
JCVI_READ_1104230166942/1009-1212
GUG.....................AGUACCCUCAUCCUCUGGCCUACCUCUGAAACGGGA...............................UAG.....................U
UUCAGGGGCUGUUUGC.G.GGUUCCGUCAGGU...GG-GGCUUGU.------UCCC.--GCC-ACUCGGGCU-CGACGCUGCGUUACCC-CGCAGGUC-A---CCGAU--
ACACGGG----UUUCGUG----GUCG-------UGAUC---UGGCUGGGG----CCGGG--CUGACCCGG-UUAGU
JCVI_SCAF_1096628073253/1767-1971
GUG.....................AGUACCCUCAUCCUCUGGCCUACCUCUGAGACGGGA...............................UAG....................-
UUUCACAGGCUGUUUGC.G.GGUUCCGUCAGGU...GG-GGCCUGUU.----.CCCC---UCC-ACUCGGGCU-CGACGCUGCGUUACCC-CGCAGGUC-A---CCGAU--
ACACGGG----UUUCGUG----AUCG-------UGAUC---UGGCUGGGA----CCGGG--CUGAUCCGG-UCAGU
JCVI_SCAF_1096626671002/541-746
UUG..................GUGAGUACCCUCAUCCUCUGGCCUACCUCUGGAACGGGA...............................UAG-
....................UUUCACAGGCUGUUUGC.G.GGUUCCGUCAGGC...GA-GGCAUG-------GCCUCU--CCC-ACUCGGGCU-CGACACUGCGUUACCC-
CGCAGGUC-A---CCGAU--ACACGGU----UUUCGUG----AUCG-------CGAUC---UGGUGGGAAG---CUGGA--GCGAUCCGG-CCAGU
NCBI_BBAY_READ_1106073115622/939-1144
GUG.....................AGUACCCUCAUCCUCUGGCCUACCUCUGGAACGGGA...............................UAG....................-
UUUCACAGGCUGUUUGC.G.GGUUCCGUCAGGC...GA-GGCAUG-------GCCUCU--GCC-ACUCGGGCU-CGACACUGCGUUACCC-CGCAGGUC-A---CCGAU--
ACACGGC----UUUCGUG----AUCG-------CGAUC---UGGCGGGAAA---CUGGA--GCGAUCCGG-CCAGU
JCVI_READ_1105334111736/841-1045
GUG.....................AGUACCCUCACCCUCUGGCCUACCUCAGAGACGGGA...............................UAG.....................U
UUCUCACGCUGUUUGC.G.GGUUCCGUCACGC...GA-GGCAAA-------GCCUCU--GCC-ACUCGGGCU-CGACGCUGCGUUACC--CGCAGGUC-A---GCGAU--
ACACGGG----UUUCGUG----AUCG-------UGAUC---UGGCAGGUG----CCGGA--CCGAUCCGGACCAGU
JCVI_SCAF_1096627562944/1183-1386
GUG.....................AGUACCCUCACCCUCUGGCCUACCUCAGAGACGGGA...............................UAG....................-
UUUCUCAGGCUGUUUGC.G.GGUUCCGUCAGGC...AA-GGCAUA-------GCCUCA--GCC-ACUCGGGCU-CGACGCUGCGUUACCC-CGCAG-UC-A---CCGAU--
ACACGGG----UUUCGUG----AUCG-------UGAUC---UGGCAGGUG----CCGGA--CCGAUCCGG-CCAGU
JCVI_READ_1104230091002/134-339
GUG.....................AGUACCCUCACCCUCUGGCCUACCUCAGAGACGGGA...............................UAG....................-
UUUCUCAGGCUGUUUGC.G.GGUUCCGUCAGGC...AA-GGCAUA-------GCCUCA--GCC-ACUCGGGCU-CGACGCUGCGUUACCC-CGCAGGUC-A---CCGAU--
ACACGGG----UUUCGUG----AUCG-------UGAUC---UGGCAGGGUG---CCGGA--CCGAUCCGG-CCAGU
JCVI_SCAF_1096627963782/1112-1317
UUG..................GUGAGUACCCUCACCCUCUGGCCUACCUCAGAGACGGGA...............................UAG....................-
UCUCUCAGGCUGUUUGC.G.GGUUCCGUCAGGC...GA.GGCAAC-------GCCUCU--GCC-ACUCGGGCU-CGACGCUGCGUUACCC-CGCAGGUC-A---CCGAU--
ACACGGG----UUUCGUG----AUCG-------UGAUC---UGGCAGGGUG---CCGGA--CCGAUCCGG-CCAGU
```

```
2236876002_127174/260-465
GUG..................GUGAGUACCCUCACUCCCUGGCCUACCUUGAGAACGGGA...............................UAG....................-
UUCUCCAGGCUGUUUGC.G.GGUUCCGUUAGGU......GGCGAUGUAA-AGGCC.....ACU----CGGGCU-CGACACUGCGCUACCU-CGCAGGUC-A---CCGAU--
ACACGGG----UUUCGUG----AUCG-------UGGUC--GUGUGUAGCGG--UCCGGA--CCGAUCCGG-CCAGU
JCVI_SCAF_1101668578636/1046-1249
GUG.....................AGUACCCUCACCCUCUGGCCUACCUUAAGAACGGGA...............................UAG....................-
UUCUUCAGGCUGUUUGC.G.GGUUCCGUUAGGU...GG.CGCUGUAG-..AGGCC.....ACC-----GGGCU-CGACACUGCGUUACCU-CGCAGGUC-A---CCGAU--
ACACGGG----UUUCGUG----AUCG-------UGGUC---UGUGUAGCGC---CCGGA--CCAAUCCGG-CCAGU
NZ_AUHJ01000006.1/236031-236242                GUG.....GUAACGACUAAUUCCGGGUACCCCCGGCCACUGGUCUACUGCUGUGUUGUCAU-UAUUC-
ACAGUG---GCUGUUCA......UGA................................GUCC.CGU.A-A-..---UCAACCUGC-GCCCUACU-----GGCGGGGGUUG----
AGGGCC-AGACACUGCGCUACCU-CGCAGGGCGA---CCGAU--ACACGGG---UGUUCGUG----AUCGA----UUUCCUC---CGCGGAGCUC---CCGGU--UCAUGCCGG--
----
2236876010_0627257/219-427                     AUG.....GCAAUAGCUAACGUCGAGUACCCUCGGCCUCUGGCUCACUGGAUUGAUCCGAU-UAUUC-
AAGCCA---GCUGUUUG......UGA................................GUCC.CAU.U-A-..--AUCAA---UU-UGCGCCGCAU-UGGCCAGG--UUG----
AGGGCC-AGACACUGCGCUACCU-CGCAGGGCGA----CGAU--ACACGG------UUUGUG----AUCGA---UUUUUCCC---UGAUGGAGCG----------UUGCUCCCU-
GAAGU
2236876011_053527/250-456                      AUG.....GCAACAGCUAACGUCGAGUACCCUCGGCCUCUGGCUCACUGGAUUGAUCCGAU-UAUUC-
AAGCCA---GCUGUUUG......UGA................................GUCC.CAU.A-A-..---UCAA---UU-UGCGCCGCAU-UGGCCAGG--UUG----
AGGGCC-AGACACUGCGCUACCU-CGCAGGGCGA----CGAU--ACACGG------UUUGUG----AUCGA----UUUUCCC---UGAUGGAGCG---UCCCU--CCGUGGGGC--
----
NZ_DS999233.1/8449-8663                        AUG.....GCAACAGCUAACGUCGAGUACCCUCGGCCUCUGGCUCACUGGAUUGAUCCGAU-UAUUC-
AAGCCA---GCUGUUUG......UGA................................GUCC.CAU.A-A-..---UCAA---UU-UGCGCCGCAU-UGGCCAGG--UUG----
AGGGCC-AGACACUGCGCUACCU-CGCAGGGCGA----CGAU--ACACGG------UUUGUG----AUCGA---UUUUUCCC---UGAUGGAGCG---UUGCUCCCUGAAGUGG-
AAAGC
TDF_OR_ARG04_113m_c1102684/373-592             UUG.....................AGUACCCUCGAUCUCUGGCCCACUGUCCCGAUACGAU-
UAUUCGGGGUCA---GCUGUUUG.......UAA................................GUCC.CGU.A-U-..---UCAACUUGU-GCCGCACAG----GGCAGG--
UUG----AGGGCC-AGACACUGCGCUAUCC-CGCAGGGCGA---CCGAU--ACACGGG---UGUUCGUG----AUCGUA-UAUCCCCC-C-AAGGAGCGUCU--GCGGGG--
UUGAUCCCGGGCAGU
SA_S2_NOR15_50m_c10531239/220-423              AUG..................UUGAGUACCCUCGACCUCUGGCUCACUGCAACAAUACGAU-UAUUU-
GUUGCA---GCUGCUCGU.....UAG.................................UCC.CGU.A-U-..---UUAA---CC-UGCGUCGUAUUGAACAGG---UUA----
AGGGCC-AGACACUGCGCUACCC-CGCAGGGCGA---CCGAU--ACACUUC---AGUUCGUG----AUCGU---AAUUCCCCU-UUGGAGCGUC---------------------
CCAGC
SA_S1_NOR08_45m_c10836286/193-384              AUG..................UUGAGUACCCUCGACCUCUGGCUCACUGUGGUAAUGCGAU-UAUUU-
ACUACA---GCUGUUUGU.....UAG.................................UCC.CGU.A-U-..---UUAA---UC-UGAGUCGCAUUGACCAGA---UUA----
AGGGCC-AGACACUGCGCUACCC-CGCAGGGCGA---CCGAU--ACACACC---AGUGAGUG----AUCGUA--AUUCCCCC---U------------------------------
--AU
#=GC SS_cons                                   ::<..................<<<<<____>>>>>>,,,,,,,,,,,,,-
,,,,,,.____..............................,,....................,,,,,,,,,,,,,<,<<.<.<.,--....-
```

```
..<<<,..<<.<<<,______...>>>>>.-.>>>..-->>>>,>-,,..((((<<______.>><<<<<<--...-<<<<..-<<<<__....___>>>>....>>>>......-
->>>>...>>,,,,,,,,...<<<<<..____>>>>>.,))))
#=GC R2R_LABEL
sss.......................................b...............................................ettt-
..............................+1.......8.........9...<.>.....................................02.....................
..........................................................f.........l................45....
#=GC R2R_XLABEL_ticks
.S..........................................................................................T.......................
................................................................................J...................................
......................................................................
#=GF R2R outline_along_backbone t rgb:0,0,0
#=GF R2R outline_along_backbone s rgb:0,0,0
#=GF R2R tick_label ticks:T Stop
#=GF R2R tick_label ticks:S Start
#=GF R2R var_backbone_range 0 2
#=GF R2R var_backbone_range b e
#=GF R2R var_backbone_range f l
#=GF R2R var_backbone_range 4 5
#=GF R2R var_backbone_range < >
#=GF R2R var_backbone_range - +
#=GF R2R var_backbone_range 8 9
#=GF R2R multistem_junction_bulgey ticks:J J0 -45 1 0 0 0 -90 J1 -45 1 0 0 0 -90 J2 -45 1 0 3 0 -90
//
```

File S3. Sequence alignment of the *ilvB* motif.

```
# STOCKHOLM 1.0
NZ_AOGY02000056.1/38100-38241
AUGCCCGUUCUUGGAUUGUCUAGCUAUGUCCUCUCCACUCUCGUCCUAAUCGUCGUCGUAGUCUCCUCAUACAUACGUAGCUUUCUGUCAUAAUCUCC.CUUCGUUUCCUCGGUCG
ACAGAAAGCUGAAGUCGACGGGGAAACCAAAU..UGGGAGGAU.CUUAUG
NZ_AOGW02000016.1/102046-101915
AUGCCCGUUCUUGGAUUGUCUAGUUAUGUCCUCUCUACUCUC.........GUCGUCGUAGUCUCCUCAUACAUACGUAGCUUUCUGUCAUAAUCUUC.CUUAGUUUCCUCGGUCG
ACAGAAAGCAAA.GUCGACGGGGAAACCAAAU..UGGGAGGAU.CUUAUG
NZ_AOGZ02000014.1/1080822-1080682
AUGCCCGUUCUUGGAUUGUCUAGCUAUGUCCUCUCUACUCUCGUCCUAGUCGUCGUCGUAGUCUCCUCAUACAUACGUAGCUUUCUGUCAUAAUCUCC.CAUCGUUUCCUCGGUCG
ACAGAAAGCAAG.GUCGACGGGGAAACCACAC.AGGAGAGGAUCUUUAUG
NZ_ANIL01000007.1/102533-102393
AUGCCCGUUCUUGGAUUGUCUAGCUAUGUCCUUUCUACUCUCGUCCUAGUUGUCGUCGUAGUCUCCUCAUACAUACGUAGCUUUCUGUCAUAAUCUCC.CAUCGUUUCCUCGGUCG
ACAGAAAGCAAG.GUCGACGGGGAAACCAACUAAGGAGAGGUUCGUUAUG
NZ_AKXE01000002.1/2159191-2159331
AUGCCCGUUCUUGGAUUGUCUAGCUAUGUCCUCUCUACUCUCGUCCUAGUUGUCGUCGUAGUCUCCUCAUACAUACGUAGCUUUCUGUCAUAAUCUCC.CAUCGUUUCCUCGGUCG
ACAGAAAGCAAG.GUCGACGGGGAAACCAACCAAGGAGAGGAUCUGUAUG
NZ_AOGX02000015.1/1216994-1217130
AUGCCUUCU...............UAUACCCUCAUGCUUGUCGUC......CUCGUCGUCGUCUCCCCAUACAUACGUAGCUUUCUGUCAUAAUCCUCACUUCGUUUCCUCGGUCG
ACAGAAAGCAUCAGUCGACGGGGAAACCACUA..GAGGAGACAGGUUAUG
NC_010842.1/816626-816761
AUGCCUUCU...............UAUACCCUUACCACUGUCGUC......CUCGUCGUCGUCUCCUCAACCAUACUUAGCUUUCUGUCAUAAUCUUC.UUUAGUUUCCUCGGUCG
ACAGAAAGCAAAUGUCGACGGGGGAACCAUCA..AAGGAGACAUGUUAUG
#=GC SS_cons
:::::::,,,,,,,,,,,,,,,,,,,,,,,,,,,,,,,,,,,,,,,,,,,,,(((,<<<<_____________>>>>,,<<<<<<<<<<---------.--<<<______>>>--
>>>>>>>>>>,,,.,,))):::::::::::::::::::::::::::::::::
#=GC R2R_LABEL
sss.c....1.............2..a...UUU......UUU.........UUUUUUUUUUUU.........UUU......UUUUUU...ttt.......................
.........................b........rrrrrr.......mmm
#=GC R2R_XLABEL_ticks
.S.............................U........U...........U.J......U...........U........U..U.....T........................
.....................................R..........M.
#=GF R2R outline_along_backbone t rgb:0,0,0
#=GF R2R outline_along_backbone s rgb:0,0,0
#=GF R2R outline_along_backbone m rgb:0,0,0
#=GF R2R outline_along_backbone r rgb:0,0,0
#=GF R2R var_backbone_range 1 2
#=GF R2R outline_nuc U
```

```
#=GF R2R tick_label ticks:T Stop
#=GF R2R tick_label ticks:S start
#=GF R2R tick_label ticks:M Main ORF
#=GF R2R tick_label ticks:R RBS
#=GF R2R set_dir pos0 90
#=GF R2R place_explicit a a-- -45 1 0 0 0 -90
#=GF R2R place_explicit b b-- -45 1 0 0 0 -90
#=GF R2R multistem_junction_circular_solver ticks:J s0 0 ai s1 -90 ai s2 0 ai align_stem_horiz 0 2
#=GF SORT_STATS 7 143 0
#=GF DUPLICATES NONE
//
```
